# Ferrostatin‐1‐loaded liposome for treatment of corneal alkali burn via targeting ferroptosis

**DOI:** 10.1002/btm2.10276

**Published:** 2021-12-08

**Authors:** Kai Wang, Li Jiang, Yueyang Zhong, Yin Zhang, Qichuan Yin, Su Li, Xiaobo Zhang, Haijie Han, Ke Yao

**Affiliations:** ^1^ Eye Center, The Second Affiliated Hospital, School of Medicine Zhejiang University Hangzhou China; ^2^ Zhejiang Provincial Key Lab of Ophthalmology, the Second Affiliated Hospital, School of Medicine Zhejiang University Hangzhou China; ^3^ Department of Biochemistry and Molecular Biology, School of Basic Medical Sciences Hangzhou Normal University Hangzhou China

**Keywords:** corneal alkali burn, corneal neovascularization, ferroptosis, ferrostatin‐1, liposome

## Abstract

Alkali burn is a potentially blinding corneal injury. During the progression of alkali burn‐induced injury, overwhelmed oxidative stress in the cornea triggers cell damage, including oxidative changes in cellular macromolecules and lipid peroxidation in membranes, leading to impaired corneal transparency, decreased vision, or even blindness. In this study, we identified that ferroptosis, a type of lipid peroxidation‐dependent cell death, mediated alkali burn‐induced corneal injury. Ferroptosis‐targeting therapy protected the cornea from cell damage and neovascularization. However, the specific ferroptosis inhibitor ferrostatin‐1 (Fer‐1) is hydrophobic and cannot be directly applied in the clinic. Therefore, we developed Fer‐1‐loaded liposomes (Fer‐1‐NPs) to improve the bioavailability of Fer‐1. Our study demonstrated that Fer‐1‐NPs exerted remarkable curative effects regarding corneal opacity and neovascularization in vivo. The efficacy was comparable to that of dexamethasone, but without appreciable side effects. The significant suppression of ferroptosis (induced by lipid peroxidation and mitochondria disruption), inflammation, and neovascularization might be the mechanisms underlying the therapeutic effect of Fer‐1‐NPs. Moreover, the Fer‐1‐NPs treatment showed no signs of cytotoxicity, hematologic toxicity, or visceral organ damage, which further confirmed the biocompatibility. Overall, Fer‐1‐NPs provide a new prospect for safe and effective therapy for corneal alkali burn.

## 1 INTRODUCTION

Alkali burn‐induced injury is one of the most common and devastating types of ocular injury, which could ultimately lead to blindness due to permanent visual impairment. Alkali‐induced corneal injury drastically impacts the ocular structure and visual function and is refractory to conservative treatment. Several treatment modalities, including steroids, nonsteroidal anti‐inflammatory drugs, laser photocoagulation, photodynamic therapy, fine needle vessel diathermy, amniotic membrane transplantation, and limbal stem cell transplantation, are used for alkali burns.^1^ However, there still exist numerous limitations, including poor curative effects, toxic side effects, invasive operation, and risk of graft rejection.^2^ Therefore, more efficient and safe therapy is urgently needed in the treatment of alkali burn‐induced injury.

Ocular drug delivery has always been a significant pharmaceutical challenge due to the complex physiology and anatomy of the ocular surface.^3^, ^4^ Structural variation of different layers of cornea can pose a barrier following drug administration by any route.^5^ Due to the lower toxicity, improved biorecognition, and enhanced drug bioavailability, a variety of nanocarriers have been intensively investigated as possible carriers for delivering therapeutic agents to treat refractory disorders, including cancer,^6^, ^7^, ^8^ spinal cord injury,^9^ brain diseases,^10^ and corneal diseases.^11^, ^12^, ^13^ Since the mid‐1990s, plenty of liposome formulations combined with drugs or biologics, including liposomal doxorubicin, cytarabine, vincristine, irinotecan, and amphotericin B, have been approved by the Food and Drug Administration for clinical use.^14^ Liposomes are unilamellar or multilamellar phospholipid vesicles, where the loaded hydrophobic and hydrophilic agent can be encapsulated within the phospholipid bilayer and the aqueous core, respectively.^15^, ^16^ Drugs with high toxicity or low bioavailability benefit from the improved biocompatibility and stabilizing nature of liposomes, which enables the safety and efficacy of drugs. Although the cornea hinders deep ocular drug permeation as a physiological barrier, liposomes have been shown to circumvent this limitation and achieve sustained delivery of the drug into both the anterior and posterior segments.^17^, ^18^ Therefore, the liposome could serve as a potential nanocarrier for ocular drug delivery to repair the injured cornea.

Numerous studies have demonstrated that inflammatory response and corneal neovascularization (CNV) play pivotal roles in corneal injury and vision loss.^19^, ^20^, ^21^ In the alkali‐burned cornea, the wound‐healing response could lead to a dysregulated inflammatory response. Various cells, such as inflammatory, stromal, and epithelial cells, are involved in these processes. Meanwhile, the angiogenesis factors are also activated.^22^ Alkali burn‐induced conjunctival and corneal epithelium injuries may also cause a limbal stem cell deficiency, leading to opacification and neovascularization of the cornea.^23^

Further, it has been well documented that alkali burns promote the accumulation of reactive oxygen species (ROS) in the cornea.^24^, ^25^, ^26^, ^27^ Following a severe corneal injury, oxidative stress could lead to an antioxidant/prooxidant imbalance in corneal cells. The insufficiently scavenged ROS greatly contribute to excessive intracorneal inflammation, scar formation, and CNV. Moreover, the increased oxidative stress could cause disruption of mitochondrial permeability transition pore, perturbation of electron transfer, and destruction of cell membranes, ultimately leading to cell death.^28^ Taken together, oxidative stress, inflammation, and neovascularization collectively aggravate the corneal injury and lead to vision loss. Previous studies indicated that ROS‐related oxidases play a role in corneal alkali burn.^24^ In addition, the inhibition of alkali‐induced oxidative stress appeared to ameliorate corneal inflammation as well as CNV and promote corneal healing.^27^, ^29^ These results suggest that oxidative stress may represent a potential therapeutic target for corneal alkali burn. However, the underlying mechanisms are poorly understood.

Ferroptosis is a novel type of programmed cell death characterized by the lethal accumulation of lipid peroxides. It is biochemically, morphologically, and genetically different from other established forms of regulated cell death, such as apoptosis, autophagy, and necroptosis.^30^, ^31^ Ferroptosis is associated with numerous pathological processes, including tumorigenesis, neurodegenerative diseases, ischemia/reperfusion (I/R) injury, and liver injury.^32^, ^33^, ^34^ Notably, it has been suggested that ferroptosis may contribute to the pathogenesis of some ocular diseases, including retinal pigment epithelium dysfunction, defects in corneal endothelial cells, and photoreceptor degeneration.^35^, ^36^, ^37^, ^38^ Nevertheless, the precise role of ferroptosis in the development of corneal injury induced by alkali burn remains unclear.

In this study, we identified that ferroptosis plays a vital role in alkali burn‐induced corneal injury. Mechanistically, severe injury results in the accumulation of ROS, up‐regulation of the ferroptosis promoter, and down‐regulation of the ferroptosis depressor, which facilitates lipid peroxidation and mitochondria disruption, and eventually triggers ferroptosis. Targeting ferroptosis by specific inhibitor ferrostatin‐1 (Fer‐1) protects corneal cells from damage and angiogenesis, therefore promoting the corneal healing processes. However, as a promising pharmacological molecule, the poor solubility of Fer‐1 in aqueous media raises a concern regarding the clinical application of Fer‐1 in corneal diseases. A plethora of studies have demonstrated the effectiveness of liposomes for cornea drug delivery applications. Due to the biodegradable and biocompatible lipid assemblies, liposomes could achieve high solubility and drug‐loading, enhanced precorneal retention time, increased transmembrane permeation and absorption, as well as sustained release of the drug along with low toxicity.^14^ Therefore, liposomes possess a substantial potential for clinical applications. In light of this, we designed and prepared Fer‐1‐loaded liposome (Fer‐1‐NPs) to treat corneal alkali burn via targeting ferroptosis. Interestingly, Fer‐1‐NPs exerted more curative effects regarding ferroptosis suppression, anti‐CNV efficacy as well as corneal injury protection. The Fer‐1‐NPs were expected to enhance the solubility of Fer‐1 and thereby improve its bioavailability. Moreover, the Fer‐1‐NPs showed low cytotoxicity and no damage to the cornea or visceral organs. Our findings provide solid evidence for the pathogenic mechanisms underlying alkali burn‐induced corneal injury. More importantly, we prepared Fer‐1‐NPs for safely and effectively inhibiting ferroptosis and CNV, which may provide potential targets for the development of novel therapeutic strategies.

## 2 MATERIALS AND METHODS

### 2.1 Materials

Fer‐1 was obtained from MedChemExpress. Dexamethasone (Dex) was purchased from Meilunbio. Soybean lecithin, cholesterol, and 1,2‐distearoyl‐sn‐glycero‐3‐phosphoethanolamine‐N‐[methoxy (polyethylene glycol)‐2000] (DSPE‐mPEG) were obtained from AVT Pharmaceutical Technology. Green fluorescent protein‐transduced human umbilical vein endothelial cells (HUVEC‐GFP) were obtained from Beyotime. Human corneal epithelial cells (HCECs) were obtained from the ATCC. Calcein‐AM/PI double stain kit was purchased from Yeasen. A Cell Counting Kit‐8 (CCK‐8) was purchased from Dojindo. Recombinant human vascular endothelial growth factor (rhVEGF) was purchased from Peprotech. Matrigel Matrix Growth Factor Reduced was obtained from BD Bioscience. Rabbit anti‐4 hydroxynonenal (4‐HNE), anti‐glutathione peroxidase 4 (GPX4), anti‐acyl‐CoA synthetase long‐chain family member 4 (ACSL4), anti‐CD31, and anti‐α‐smooth muscle actin (α‐SMA) antibodies were purchased from Abcam. Rabbit anti‐IL‐1β and anti‐IL‐6 antibodies were purchased from Affinity Bioscience. PrimeScriptTM RT Master Mix was acquired from Takara. ChamQ SYBR qPCR Master Mix was acquired from Vazyme.

### 2.2 Liposomes preparation and characterization

Liposomes were synthesized using a thin‐film hydration method.^39^, ^40^, ^41^ Specifically, in a round‐bottom flask, 2 mg of Fer‐1 (or 3 mg Dex), 8 mg of cholesterol, 10 mg of DSPE‐mPEG, and 30 mg of soybean lecithin (molar ratio, 2:5:1:10) were added to 10 ml of methylene chloride and dissolved completely. The mixture was evaporated using a rotary evaporator under reduced pressure until a homogeneous phospholipid film formed. The obtained phospholipid film was then hydrated with 10 ml of ultrapure water for 1 h. Subsequently, the mixture was ultrasonicated with 50 W for 2 min at 4°C (Biosafer). Homogeneous Fer‐1‐NPs and Dex‐NPs were obtained by centrifugation (Thermofisher) at 6000 rpm for 10 min and filtration with a 0.22 μm‐filter (Millipore). It is worth noting that high‐speed centrifugation and filtration were used to separate the nonencapsulated insoluble drug and sterilization, respectively, in this study. The hydrophobic fluorescent dye Nile Red loaded liposomes (Nile Red‐NPs) were prepared using the same procedure. All the prepared liposomes were stored at 4°C for further use.

The size (hydrodynamic diameter) and polydispersity index (PDI) of the nanoparticles were measured by dynamic light scattering (DLS). In detail, liposomes were first filtered through a 0.22‐μm filter unit (Millipore) and then measured using Zetasizer Nano‐ZS (Malvern). The morphology of Fer‐1‐NPs was characterized with a cryo‐transmission electron microscope (cryo‐TEM, FEI) operated at an accelerating voltage of 200 kV. The cryo‐TEM size of the nanoparticle was quantified using ImageJ software (NIH).

To evaluate the concentration and encapsulation efficiency (EE) of the drugs, the nonencapsulated Fer‐1 (Dex) was collected by ultrafiltration centrifugation (6000 rpm, 10 min) and quantified using high‐performance liquid chromatography (HPLC) in triplicate. The following HPLC conditions for Fer‐1 (Dex) were used: A reverse C18 column (75 mm × 4.6 mm, 3.5 μm, Agilent), mobile phase consisting of 0.1% formic acid in a linear gradient of acetonitrile:water (v:v) from 50:50 to 95:5 (from 20:80 to 95:5 for Dex), injection volume of 20 μl, flow rate of 1.0 ml/min, and detection wavelength of 254 nm (240 nm for Dex). The weight of Fer‐1 (Dex) was calculated according to the standard curves (Figure S1) generated from different concentrations of drug solutions. The EE was calculated according to the following equation: EE (%) = (the weight of total drug − the weight of free drug)/the weight of total drug × 100%. The samples were diluted to a final drug concentration of 200 μM according to the tested concentration by HPLC.

### 2.3 In vitro drug release

Drug release profile was explored in vitro using dynamic dialysis methods. Briefly, 8 ml of free Fer‐1 or Fer‐1‐NPs were filled into a dialysis bag (MWCO 3500 Da) and immersed in 30 ml of 1% sodium dodecyl sulfate solution as release medium at 37°C. An aliquot of 1 ml release medium was extracted at 10 min, 20 min, 30 min, 1 h, 3 h, 6 h, 9 h, 12 h, 24 h, 36 h, and 48 h, and then an equal volume of the dissolution medium was immediately supplemented. The extracted medium was freeze‐dried and then redissolved in 0.4 ml methanol. Finally, the amount of released Fer‐1 was determined by HPLC in triplicate. The cumulative release rates were normalized.

### 2.4 Cell culture

HUVEC‐GFP were cultured in DMEM (Corning); HCECs were cultured in DMEM/F‐12 (Corning). All the media were supplemented with 10% FBS, 1% penicillin/streptomycin unless specifically stated. Cells were maintained at 37°C and 5% CO_2_ in a humidified incubator.

### 2.5 Cellular uptake

HCECs were seeded at a density of 1.5 × 10^5^ cells/well in a 12‐well plate. After culturing for 24 h, the cells were incubated with free Nile Red or Nile Red‐NPs for different time points (5 min, 15 min, 30 min, 1 h, 2 h, and 4 h). Both treatments contained identical final concentration of Nile Red (5 μM). The experiment was stopped by rinsing the cells three times with phosphate‐buffered saline (PBS) to remove nonintracellular Nile Red. Finally, the HCECs were photographed by fluorescence microscope (Leica). The cellular uptake efficiency was evaluated by the fluorescence intensity of Nile Red within cells.

### 2.6 Angiogenesis assays

#### 2.6.1 Scratch wound‐healing migration

HUVEC‐GFP were seeded at a density of 5 × 10^5^ cells/well in a 6‐well plate. The confluent cell monolayers were then scratched using a sterile 200‐μl micropipette tip and washed with sterile PBS. Subsequently, the cells were incubated for 6 h with 2 ml of fresh medium containing 1.5% FBS and divided into four groups as follows: (1) no treatment (control group); (2) treated with rhVEGF only (at a final concentration of 200 ng/ml); (3) treated with rhVEGF (at a final concentration of 200 ng/ml) and Fer‐1 (at a final concentration of 5 μM); (4) treated with rhVEGF (at a final concentration of 200 ng/ml) and Fer‐1‐NPs (at a final Fer‐1 concentration of 5 μM). HUVEC‐GFP were examined at 0 and 6 h using fluorescence microscope, and the scratch widths were quantified using ImageJ software. The healing rate was calculated according to the following formula: (0 h scratch width − 6 h scratch width)/0 h scratch width. All healing rates were normalized to the control group.

#### 2.6.2 Matrigel tube formation

A 24‐well plate was pre‐chilled with 150 μl of Matrigel per well and then allowed to solidify at 37°C for 30 min. HUVEC‐GFP at a density of 5 × 10^4^ cells/well were suspended in 500 μl of basal medium and divided into four groups as follows: (1) no treatment (control group); (2) treated with rhVEGF only (at a final concentration of 200 ng/ml); (3) treated with rhVEGF (at a final concentration of 200 ng/ml) and Fer‐1 (at a final concentration of 5 μM); (4) treated with rhVEGF (at a final concentration of 200 ng/ml) and Fer‐1‐NPs (at a final Fer‐1 concentration of 5 μM). Next, the mixture was gently added into the polymerized Matrigel. After 6 h of incubation, images were captured under fluorescence microscope, and tube formation was quantified using the angiogenesis analyzer tool of ImageJ software. All numbers of junctions were normalized to the control group.

### 2.7 Cytotoxicity assays

#### 2.7.1 CCK‐8 assay

HCECs were plated at a seeding density of 1.5 × 10^4^ cells/well in a 96‐well plate with 90 μl of basal medium overnight. Then, sterile PBS and Fer‐1‐NPs were supplemented into each well to reach the final Fer‐1 concentrations of 0, 0.1, 1, 2, 5, 10, 15, and 20 μM, respectively. Following 24 h of incubation, 10% CCK‐8 solution was supplemented and incubated for an additional 2 h. After that, cell viability was evaluated by recording the absorbance at 450 nm using microplate reader (Bio‐Rad iMark).

#### 2.7.2 Live/dead assay

HCECs were seeded at a density of 1.5 × 10^5^ cells/well in a 12‐well plate with 900 μl of basal medium overnight. Subsequently, sterile PBS and Fer‐1‐NPs were added into each well to reach the final Fer‐1 concentrations of 0, 1, 5, 10, and 20 μM, respectively. Following another 24 h of incubation, HCECs were washed and co‐stained with calcein‐AM (at a final concentration of 0.67 μM) and propidium iodide (at a final concentration of 50 μg/ml) for 30 min. Next, the green (live) or red (dead) cells were observed and photographed with fluorescence microscope.

### 2.8 Alkali burn‐induced corneal injury in mouse model

The research complied with the Association for Research in Vision and Ophthalmology Statement and the Use of Animals in Ophthalmic and Vision Research for all animal experiments. All mice studies were approved by the Zhejiang University Administration on Laboratory Animal Care. C57BL/6 mice (male, 6–8 weeks) were purchased from Shanghai SLAC Laboratory Animal Co., Ltd.

The animal model of alkali burn‐induced corneal injury was established as previously reported.^21^ In brief, the mice were anesthetized with a sodium pentobarbital (100 mg/kg) intraperitoneal injection and proparacaine (0.5%) topical application to the right eye. After that, a 2 mm‐diameter round filter paper pre‐soaked into 1 M NaOH was blotted for 5 s and attached to the center of the right cornea for 30 s. Subsequently, the ocular surface was immediately washed using 10 ml saline for 60 s.

### 2.9 Precorneal retention evaluation

The alkali‐burned mice were anesthetized and topically instilled with 10 μl of free Nile Red (50 μM) or Nile Red‐NPs (50 μM) before imaging. The head region was imaged using an IVIS Lumina imaging system (PerkinElmer) equipped with filter sets (excitation/emission, 535/600 nm). Imaging was performed at different time points (every 5 min) up to 30 min after administration. The initial fluorescence intensity was set as 100%, and that at following time points were normalized accordingly.

### 2.10 Clinical assessment

Sixty mice were randomly divided into six groups. In addition to healthy mice without alkali burn, the alkali‐burned mice were treated with saline solution, Fer‐1 solution (200 μM, in 1% v/v DMSO/saline), Fer‐1‐NPs (200 μM), dexamethasone solution (Dex, 200 μM, in 1% v/v DMSO/saline), or Dex‐NPs (200 μM). In each group, eye drops were administered (10 μl, twice a day) in the injured eyes for 14 consecutive days. Before administration (day 1) and after administration (days 3, 7, and 14), slit‐lamp examinations were performed to evaluate the corneas under systemic anesthesia. The corneal injury score was assessed according to the established method^21^:

1. Corneal opacity was graded with a score of 0 to 4, where 0 = clear cornea; 1 = slight haze, pupil visible; 2 = moderate opacity, but pupil detectable; 3 = severe opacity, pupils hardly detectable; and 4 = complete opacity, pupil undetectable.
2. CNV was graded with a score of 0 to 3, where 0 = no CNV; 1 = CNV within the corneal limbus; 2 = CNV over the corneal limbus to the corneal center; and 3 = CNV within the corneal center.
3. Neovessel size was graded with a score of 0 to 3, where 0 = no detectable neovessels; 1 = neovessels detectable under microscope; 2 = neovessels easily seen under microscope; and 3 = neovessels easily seen without microscope.

If cornea perforation occurred, the total clinical assessment score was rated as 10. CNV was calculated as follows: S = C/12 × 3.1416 × [R^2^ − (R − L)^2^]. In this formula, S represented the CNV area, C represented the clock hour points covered by the CNV, R represented the radius of the cornea, and L represented the longest length of the neovessel.

### 2.11 Histological and immunohistochemical assessment

On day 14, the right eyes were dissected and fixed in 4% paraformaldehyde (Biosharp) overnight. Then, the eyes were washed in PBS and embedded in paraffin. Histological sections (4 μm) were stained with hematoxylin–eosin (H&E) or immunostained with 4‐HNE, Acsl4, Gpx4, α‐Sma, and CD31, separately. All stained sections were examined and photographed using an Olympus BX‐61 microscope. The central corneal thickness and the average optical density (AOD) were quantified by ImageJ software.

### 2.12 Immunofluorescent staining

After 14‐day treatment, the right eyes were dissected, fixed, embedded, and sectioned. Then, paraffin‐embedded 4‐μm sections were deparaffinized and rehydrated in successive baths of xylene and ethanol, followed by heat‐induced epitope retrieval. After blocking with 5% bovine serum albumin/PBS for 1 h, IL‐1β and IL‐6 antibodies were added into the blocking buffer at 4°C overnight. Secondary antibodies were then incubated in the blocking buffer at room temperature for 1 h. Afterward, nuclei were stained with 4,6‐diamidino‐2‐phenylindole, and slides were mounted with Fluorescent Mounting Medium (Dako). Sections were analyzed using a fluorescence microscope (Olympus BX‐63), and the AOD was measured using ImageJ software.

### 2.13 Measurement of corneal ROS production

Corneal ROS stress production was measured using CellROX Green reagent (Thermo Fisher) as described in the previous study.^24^ NADPH oxidase inhibitor Diphenyleneiodonium Chloride (DPI, Selleck) was added for comparison. In brief, after alkali burn and 14‐day treatment, the fresh corneal flat‐mounts were dissected, washed with PBS, and permeabilized in 0.5% Triton‐X for 10 min. Immediately, 5 μM CellROX reagent was added to the sections followed by incubation for 30 min at 37°C. Following incubation, the flat‐mounts were washed three times with PBS. Fluorescence signals of ROS were detected with excitation and emission wavelengths at 485/530 nm under a fluorescence microscope (Olympus BX‐63), and the AOD was measured using ImageJ software.

### 2.14 Mitochondrial membrane potential (ΔΨm) detection

ΔΨm was measured using the sensitive fluorescent probe JC‐1, a cationic dye of 5,5′,6,6′‐tetrachloro1,1′,3,3′‐tetraethyl benzimidazol carbocyanine iodide.^42^ Briefly, the eyes were removed 14 days after treatment, and were immediately frozen in OCT compound (Sakura Finetek). The OCT‐embedded slices of the cornea were prepared at 6 μm thickness. Afterward, the cornea slices were stained with 1× JC‐1 (YEASEN) at 37°C for 30 min in a dark place. The stained slides were washed three times with JC‐1 buffer and then observed immediately with the fluorescence microscope (Olympus BX‐63), and the AOD was measured using ImageJ software.

### 2.15 Quantitative real‐time polymerase chain reaction

On day 14, the mice were sacrificed and the right corneas were enucleated. Total RNA was extracted using TRIzol reagent (Takara). The extracted RNA was quantified by a NanoDrop Spectrophotometer (Thermo Scientific) and reverse‐transcribed using the PrimeScript RT reagent Kit (Takara). After that, quantitative real‐time polymerase chain reaction was performed using a CFX96 Real‐Time System (Bio‐Rad) with SYBR Green Supermix (Bio‐Rad). Two corneas were combined to get one biological replica, and three biological replicas were used per group. Gene expression was normalized to *Gapdh* mRNA. The fold difference in gene expression was calculated using the comparative cycle threshold method.

### 2.16 Mitochondria evaluation

The cornea tissues were immediately fixed in 3% phosphate‐glutaraldehyde after dissection. The samples were then post‐fixed, embedded, cut, and mounted at the Electron Microscopy Core Facility of Zhejiang University. All samples were observed under a transmission electron microscope (FEI) at an accelerating voltage of 100 kV.

### 2.17 Biocompatibility evaluation in vivo

For physiological condition (without alkali burn) evaluation, 12 mice were randomly divided into four groups: no administration (healthy), treated with 10 μl of saline solution, Fer‐1 solution (200 μM, in 1% v/v DMSO/saline), or Fer‐1‐NPs (200 μM) twice daily for 14 consecutive days. On day 14, the corneas of each group were examined under the slit‐lamp microscope and were stained with sodium fluorescein to determine the integrity of the corneal epithelium. Then, the mice were sacrificed and their right eyes were dissected for H&E staining.

For pathological conditions (with alkali burn), biocompatibility evaluation was conducted in the four experimental groups (the healthy, saline, Fer‐1, and Fer‐1‐NPs groups) described in Section 2.6 (mouse model of alkali burn‐induced corneal injury). After the 14‐day treatment, blood was drawn for the white blood cells, red blood cells, hemoglobin, blood platelet, alanine transferase, aspartate transferase, blood urea nitrogen, and creatinine examinations. Five types of visceral organs, including the heart, liver, spleen, lungs, and kidneys, were excised for H&E staining.

### 2.18 Statistical analysis

All data analyses and plots were generated using GraphPad Prism (version 6.0). The differences among groups were analyzed using a student's *t*‐test or one‐way analysis of variance as appropriate. A *p* < 0.05 was considered statistically significant.

## 3 RESULTS AND DISCUSSION

### 3.1 Ferrostatin‐1 ameliorates alkali burn‐induced corneal injury in mice

**FIGURE 1:**
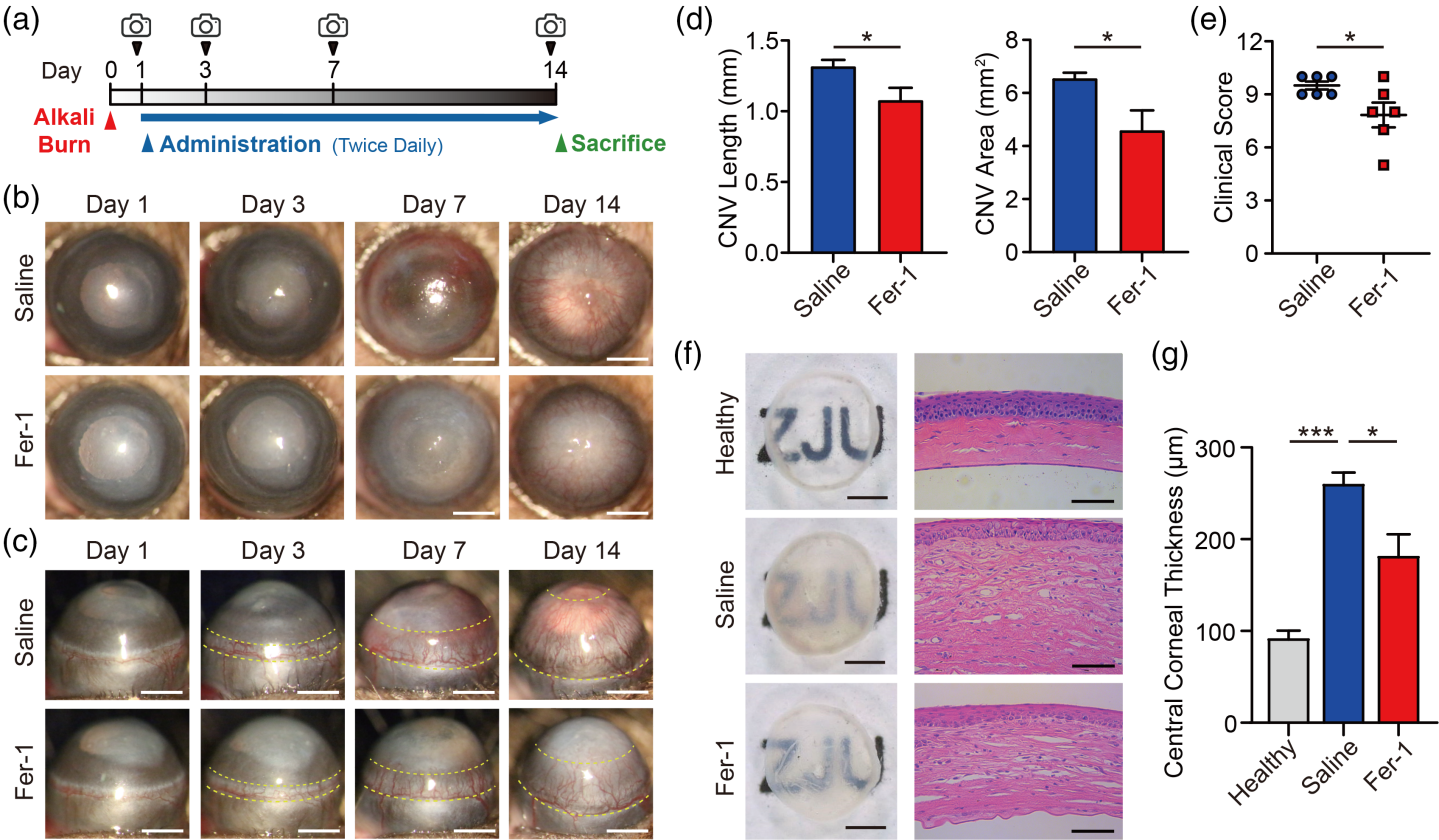
The ferroptosis inhibitor Fer‐1 ameliorates alkali burn‐induced corneal injury. (a) Schematic diagram of modeling, administration, and photograph in mice. Anterior (b) and side (c) view of the anterior ocular segment in saline‐treated and 200 μM Fer‐1‐treated groups; scale bars, 1 mm; the yellow dashed line indicates the area of CNV. CNV length, CNV area (d), and clinical assessment score (e) were measured in mice treated with saline or Fer‐1 (*n* = 6) on day 14. (f) Cornea images after sacrificing and dissecting on day 14 (left, scale bars, 500 μm) and hematoxylin–eosin staining images (right, scale bars, 50 μm). (g) Quantitative analyses of the central corneal thickness in the healthy (without alkali burn), saline‐treated, and 200 μM Fer‐1‐treated groups (*n* = 3). Results were presented as the mean ± *SEM*. *p < 0.05 and ***p < 0.001. Significance was calculated by Student's *t*‐test (d, e) or one‐way analysis of variance (g). CNV, corneal neovascularization; Fer‐1, ferrostatin‐1

A schematic representation of the workflow of our in vivo modeling and the assessment is provided in Figure 1a. As two main injured manifestations of alkali burn, corneal opacity, and neovascularization (CNV) were continuously examined by slit‐lamp microphotography. On day 1 before administration, the corneas of both groups were comparable with respect to opacity and neovessels, indicating a good homogeneity of modeling. In both groups, the corneal opacity aggravated over time. Similarly, the neovessels grew gradually from the limbal vascular plexus toward the corneal center by days 3, 7, and 14 (Figure 1b,c). On day 14, the Fer‐1 group showed more effective inhibition of CNV than the saline group, which was in accordance with the quantitative analyses of CNV length and area (Figure 1d). Accordingly, the clinical corneal assessment score based on CNV size, CNV area, and opacity significantly favored the Fer‐1 treatment (Figure 1e).

The gross view demonstrated that the saline‐treated‐burned cornea group exhibited obvious opacification and slight hemorrhaging, with the background pattern visually indistinguishable (Figure 1f). In contrast, the cornea in the Fer‐1 group showed a moderate opacity, and the background pattern was easier to distinguish. As for the H&E staining results (Figure 1f), the saline group exhibited prominent corneal edema, as the thickness of the epithelium and stroma increased significantly compared with the healthy group, which was also shown in the quantitative analysis of the central corneal thickness (Figure 1g). In addition, remarkable increases in inflammatory cell infiltration and CNV formation were observed in the corneal stroma. These findings may be due to the lack of structural integrity and the increased vascular permeability of the neovessels, which could result in corneal edema, inflammation, and fibrosis.^21^ In the Fer‐1 group, the edema was partially ameliorated, reflected by a statistically significant decrease in central corneal thickness compared with that in the saline group (Figure 1g). This is clinically important since, even without CNV, edema in the cornea could independently lead to a loss of vision.^43^ Although there was still evident infiltration of inflammatory cells and CNV, it was improved significantly compared to the saline group.

### 3.2 Ferroptosis is involved in alkali burn‐induced corneal injury

**FIGURE 2:**
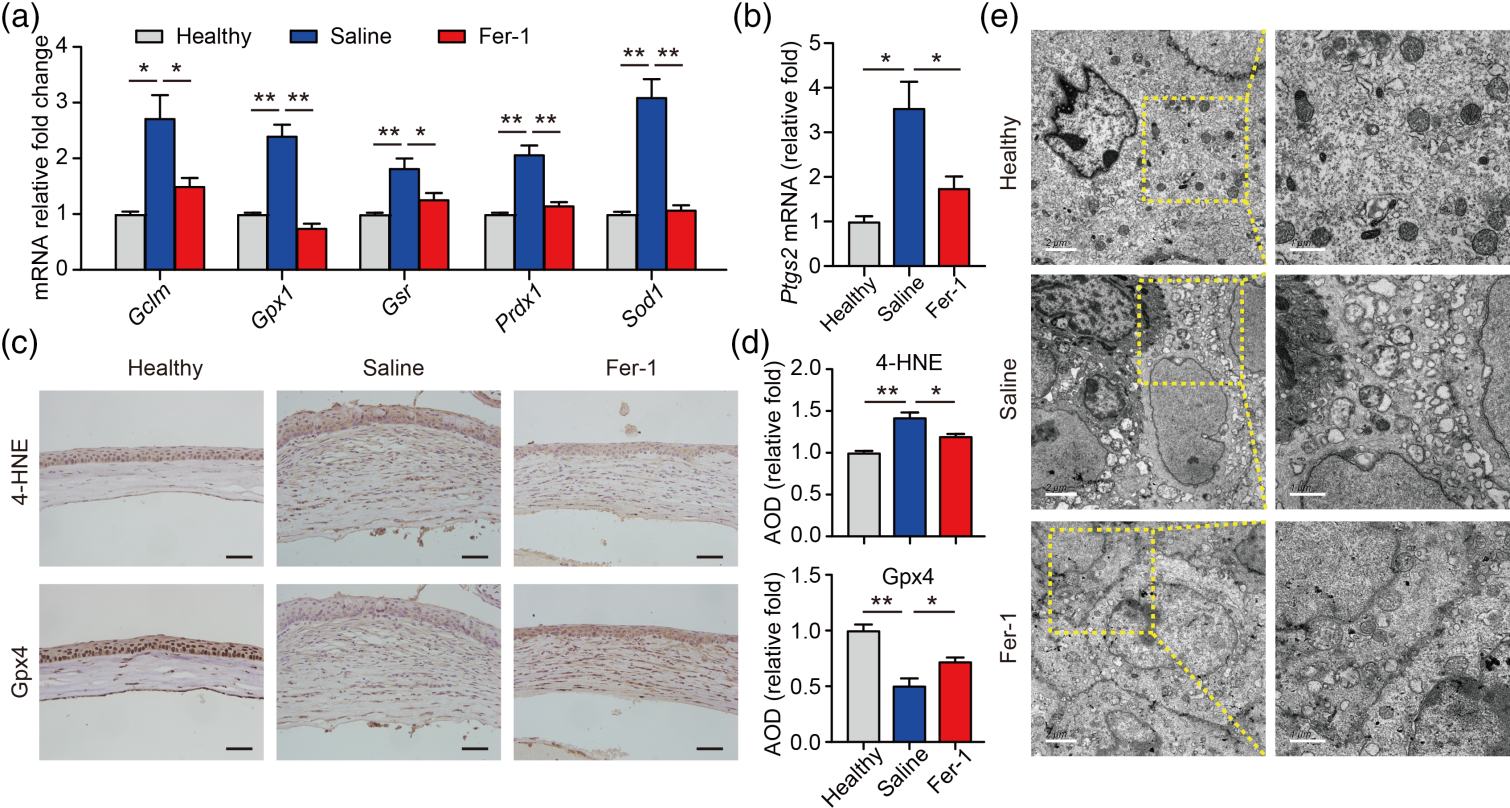
Fer‐1 protects cornea against ferroptosis in alkali burn injury. (a) Expression of the indicated reactive oxygen species‐related genes was measured using real‐time polymerase chain reaction in corneal tissue (*n* = 3). (b) Normalized *Ptgs2* mRNA were measured in the indicated groups (*n* = 3). Representative immunohistochemistry images (c) and quantitative summary (d) of corneal sections stained with 4‐HNE (top) or Gpx4 (bottom); scale bars, 50 μm; *n* = 3. (e) Transmission electron microscope images showing the morphology of mitochondria in corneal tissue obtained from the healthy, saline‐treated, and 200 μM Fer‐1‐treated groups; scale bars represent 2 μm for left images and 1 μm for right images. Results were presented as the mean ± *SEM*. *p < 0.05 and **p < 0.01. Significance was calculated by one‐way analysis of variance. AOD, average optical density; Fer‐1, ferrostatin‐1

ROS plays a pivotal role in the injury process of corneal alkali burn.^24^, ^25^, ^26^, ^27^ Kubota et al. reported the presence of ROS in alkali burn and proposed that ROS is related to the formation of CNV.^25^ Subsequent studies found that high levels of ROS in alkali burn could up‐regulate the expression levels of Nox2, Nox4, VEGF, and MMP, causing damage to the cornea. Nonselective inhibitors of NADPH oxidase could ameliorate the inflammation and CNV caused by alkali burn, suggesting that anti‐ROS can be an effective strategy for alkali burn treatment.^24^ We then detected ROS‐related genes in the corneas of the three groups. As shown in Figure 2a, the mRNA expression levels of ROS‐related *Gclm*, *Gpx1*, *Gsr*, *Prdx1*, and *Sod1* genes were significantly up‐regulated after the alkali burn, which further confirmed the increased level of oxidative stress in alkali burn. Notably, Fer‐1 administration significantly restored the expression of the above‐mentioned genes, suggesting that alkali burn‐induced elevated ROS may cause ferroptosis, and the therapeutic effect of Fer‐1 on the burned cornea may be attributed to its antagonism of ferroptosis. Therefore, we checked the corneal mRNA expression of *Ptgs2*, a key marker for ferroptosis, in the three groups. As shown in Figure 2b, compared with healthy controls, the mRNA expression level of the *Ptgs2* gene in the saline group was significantly increased. On the contrary, Fer‐1 administration significantly corrected *Ptgs2* expression compared to the saline group. *Ptgs2* is a downstream regulatory gene involved in the process of lipid peroxidation‐induced ferroptosis, the up‐regulation of which is a hallmark change of ferroptotic cell death.^44^ Our experiments demonstrated that lipid peroxidation‐dependent ferroptosis may be involved in alkali burn‐induced corneal injury.

To clarify the role of ferroptosis in corneal alkali burn, we further tested the protein levels of lipid peroxide 4‐hydroxynonenal (4‐HNE) and ferroptosis regulator Gpx4 by immunohistochemistry. Our results showed that 4‐HNE was highly expressed in the alkali‐burned cornea, accompanied by a significant decrease in Gpx4 levels, which were restored by Fer‐1 treatment (Figure 2c,d). 4‐HNE is a lipid peroxidation product after the oxidation reaction of polyunsaturated fatty acids, which could change the permeability and fluidity of the cellular membrane, leading to abnormal cell structure and function, and even cell death.^45^ Gpx4 is a peroxidase‐decomposing enzyme expressed ubiquitously that converts lipid peroxides into hydroxyl compounds, thereby protecting cell membranes from oxidative damage and inhibiting the occurrence of ferroptosis.^44^, ^46^ Taken together, our findings suggest that lipid peroxidation‐induced ferroptosis plays a key role in the process of alkali burn. The decrease of Gpx4 expression attenuated the resistance to lipid peroxidation‐triggered injury, resulting in ferroptosis of corneal cells. Moreover, Fer‐1 effectively ameliorated ferroptosis of the corneal tissue induced by alkali burn.

Emerging evidence suggests that ferroptosis represents a vulnerability caused by the incorporation of polyunsaturated fatty acids into cellular membranes, especially in the mitochondria membrane.^47^ During the occurrence of ferroptosis, accumulated lipid peroxidation could attack the mitochondria membrane, resulting in the destruction of mitochondrial membrane structure, reduced cristae, swelling, and rupture of mitochondria, which further aggravates intracellular lipid peroxidation.^48^ Previous studies have suggested that in the rat model of corneal alkali burn, ROS were elevated and mitochondrial membrane potential decreased, leading to increased cytochrome C release and dysregulation of intracellular homeostasis.^49^ To further explore the pathological mechanism of ferroptosis‐induced injury during corneal alkali burn, we examined the morphology of mitochondria in corneal epithelial cells with a transmission electron microscope. Our result showed that the corneal mitochondria in the saline group were distinctly enlarged and distorted. Moreover, these effects induced by alkali burn were partially rescued (Figure 2e). These results suggest that mitochondrial damage may be a crucial pathological mechanism in corneal alkali burn‐induced ferroptosis.

### 3.3 Preparation and characterization of the drug‐loaded liposomes

**FIGURE 3:**
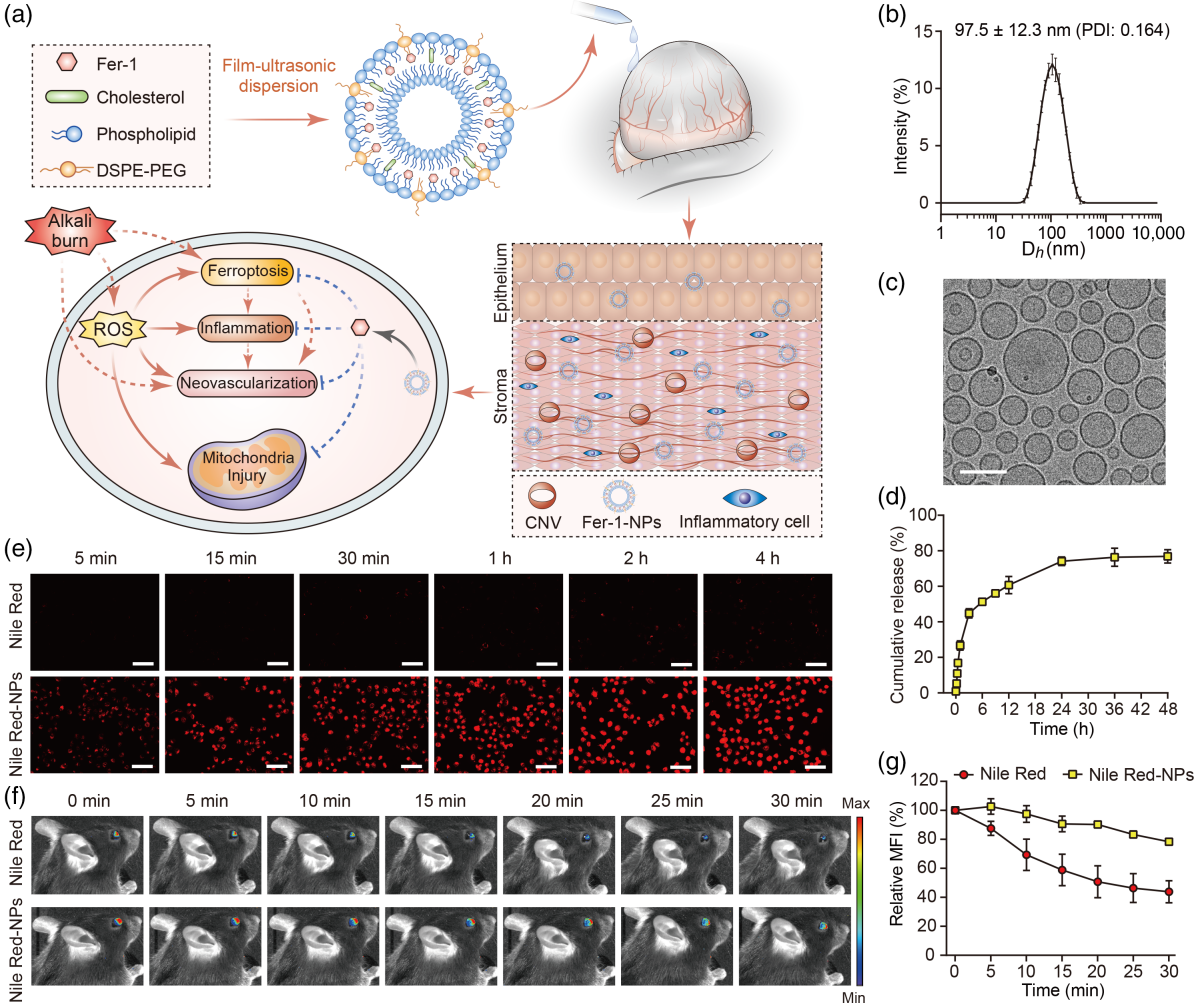
Self‐assembly and characterization of Fer‐1‐loaded liposomes and their predicted effects on corneal cells after alkali burn injury. (a) Liposomes were prepared using a thin‐film hydration method with Fer‐1 encapsulated into the hydrophobic layer. When administrated to the alkali‐burned cornea by eye drops, nanocarriers‐encapsulated Fer‐1 exhibit enhanced drug bioavailability than free Fer‐1. After the internalization of Fer‐1‐NPs, the drugs are released inside the cell, where they exert their inhibitory effects of ferroptosis, inflammation, and neovascularization. (b) The dynamic light scattering‐determined hydrodynamic diameter of the Fer‐1‐NPs (*n* = 3). (c) The cryo‐transmission electron microscope image of the Fer‐1‐NPs; scale bar, 100 nm. (d) In vitro release profiles of the Fer‐1‐NPs (*n* = 3). (e) Cellular uptake of free Nile Red and Nile Red‐NPs by human corneal epithelial cells. Scale bar, 50 μm. Representative fluorescence images of mice eyes (f) and quantification (*n* = 3) of the fluorescence signal (g) at different time points after topical administration of free Nile Red or Nile Red‐NPs. Results were presented as the mean ± SD. CNV, corneal neovascularization; DSPE‐PEG, 1,2‐distearoyl‐sn‐glycero‐3‐phosphoethanolamine‐N‐[methoxy (polyethylene glycol)‐2000]; Fer‐1‐NPs, ferrostatin‐1‐loaded liposomes; PDI, polydispersity index; ROS, reactive oxygen species

Although Fer‐1 treatment exhibited improvement effects for corneal injury to some extent, its therapeutic efficacy is limited. In terms of in vivo application, Fer‐1 is poorly soluble and rapidly cleared from the ocular surface, which leads to low bioavailability. Hence, liposomes were adopted to encapsulate Fer‐1 using a film hydration method, as illustrated in Figure 3a. Based on DLS measurements (Figure 3b), the intensity‐average hydrodynamic diameter (Dh) of Fer‐1‐NPs was 97.5 ± 12.3 nm with a PDI of 0.164. In addition, Dex‐NPs exhibited a roughly equivalent diameter of 93.1 ± 13.6 nm with a PDI of 0.238 (Figure S2). The cryo‐TEM images indicated that Fer‐1‐NPs dispersed as individual particles and had well‐defined spherical morphologies (Figure 3c). The size of Fer‐1‐NPs obtained by cryo‐TEM was 61.3 ± 23.0 nm, which was smaller than the hydrodynamic diameter measured by DLS. This is possibly due to the extensive solvation/hydration of nanoparticles in DLS measurements.^50^ The drug EE for Fer‐1‐NPs and Dex‐NPs were 95.3 ± 2.3% and 93.6 ± 3.1%, respectively. Accordingly, the concentration of Fer‐1 and Dex in liposomes was calculated to be 3.8 ± 0.1 wt% and 5.5 ± 0.2 wt%, respectively.

In vitro release profile of Fer‐1‐NPs at the predetermined time was performed as shown in Figure 3d. Approximately 5.6% and 44.8% of Fer‐1 are rapidly released from liposomes during the initial 10 min and 3 h, respectively. Then, about 74.1% and 76.3% of Fer‐1 was released at 24 h and 36 h, respectively. These results demonstrated the sustained release behavior of Fer‐1‐NPs, which could be crucial for achieving prolonged dosing intervals with enhanced drug contact.

Hydrophobic fluorescent probe Nile Red, utilized as the drug model, was encapsulated in the liposomes (Nile Red‐NPs) to investigate the cellular uptake using a fluorescent microscope. As shown in Figure 3e, strong red fluorescence from Nile Red‐NPs treated HCECs intensified with the extension of incubation time, and the internalization was largely complete within a short time (i.e., 15–30 min). In addition, cellular uptake of Nile Red‐NPs showed obviously higher fluorescence than those exposed in free Nile Red at every time point, which demonstrated the efficient internalization of Nile Red‐NPs. This behavior of fast internalization with improved cellular uptake is extremely beneficial for the administration of eye drops which have a short period of retention time.

To further validate that liposome formulation improves drug efficacy, we studied their precorneal retention by in vivo imaging technology. Eyes were imaged after topical administration of free Nile Red or Nile Red‐NPs. As shown in Figure 3f, the fluorescence intensity of the free Nile Red group rapidly decreased, with more than 50% reduction within 30 min (Figure 3g), verifying rapid and time‐dependent precorneal elimination. Conversely, Nile Red‐NPs possessed enhanced retention performance on the ocular surface. The fluorescence signal of the Nile Red‐NPs group remained stronger than that of the free Nile Red group. About 78% of fluorescence was still retained even after 30 min. These results suggested that liposome was a promising drug delivery vehicle to prolong precorneal retention time, which was beneficial for drugs with more time to permeate the cornea.

### 3.4 In vivo treatment effects of different drug formulations

**FIGURE 4:**
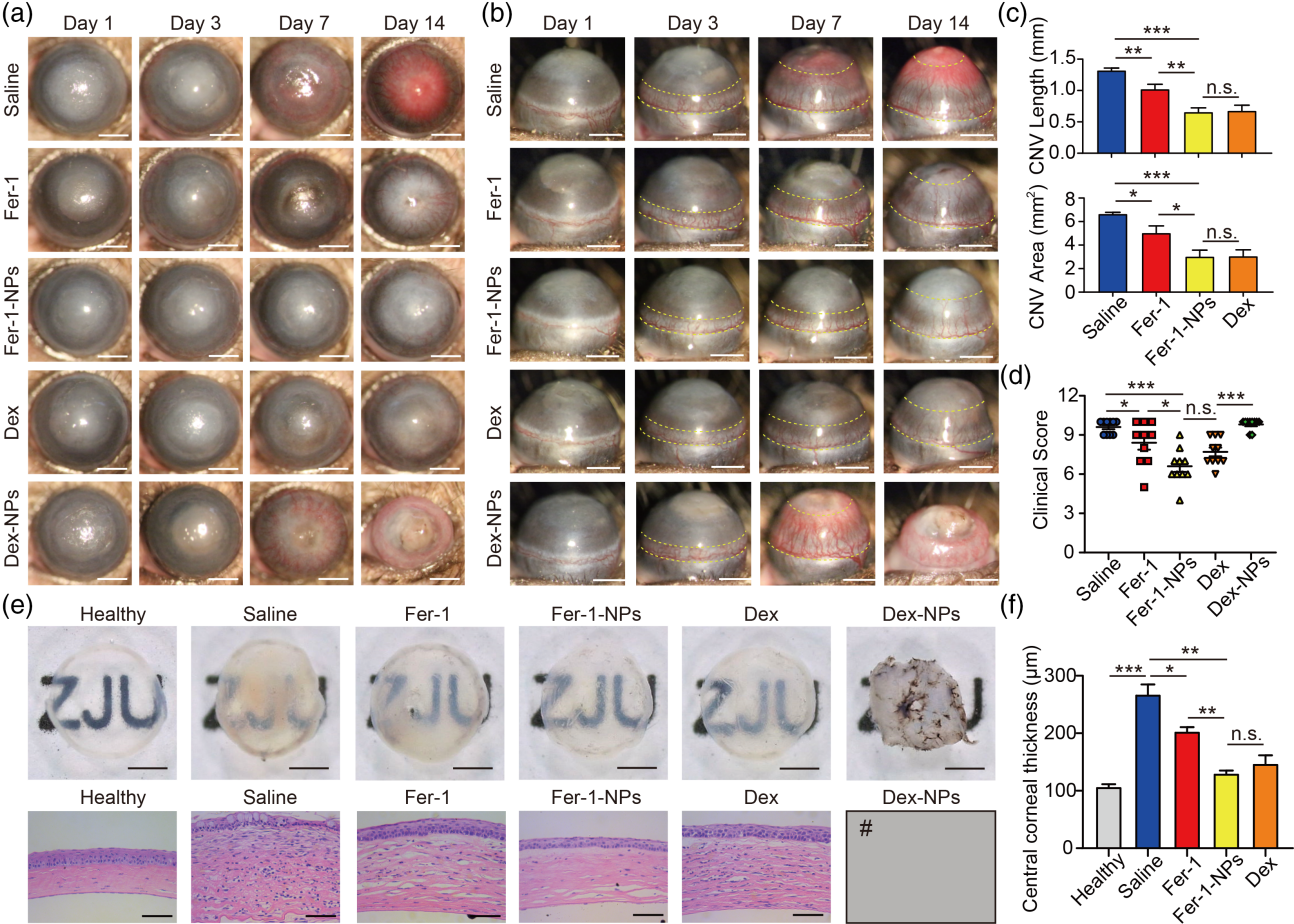
Therapeutic effects of the various treatments for the alkali‐burned cornea. Anterior (a) and side (b) view of the anterior ocular segment in saline‐treated, 200 μM Fer‐1‐treated, 200 μM Fer‐1‐NPs‐treated, 200 μM Dex‐treated and 200 μM Dex‐NPs‐treated groups; scale bars, 1 mm; the yellow dashed line indicates the area of CNV. CNV length, CNV area (c), and clinical assessment score (d) were measured in the indicated groups (*n* = 10) on day 14. (e) Cornea images after sacrificing and dissecting on day 14 (top, scale bars, 500 μm) and hematoxylin–eosin (H&E) staining images (bottom, scale bars, 50 μm). (f) Quantitative analyses of central corneal thickness in the indicated groups (*n* = 3). ^#^The Dex‐NPs‐treated group is not included in the CNV measurements, H&E staining images, and central corneal thickness analyses since most corneas in this group presented perforation and thus were not applicable for analyses. Results were presented as the mean ± *SEM*. *p < 0.05, **p < 0.01, ***p < 0.001, and n.s. stands for not statistically significant. Significance was calculated by one‐way analysis of variance. CNV, corneal neovascularization; Fer‐1‐NPs, ferrostatin‐1‐loaded liposomes

We treated alkali‐burned corneas with different drug formulations and investigated the therapeutic effects. As shown in Figure 4a,b, in the saline group, CNV almost covered the whole cornea, and the central pupil region was opaque and hyperemic on day 14. By quantifying the angiogenesis and degree of injury, the mean CNV length, CNV area, and clinical assessment score were 1.31 mm, 6.61 mm^2^, and 9.6 (Figure 4c,d), respectively. The Fer‐1 group showed moderate inhibition of neovascularization, with a mean CNV length of 1.02 mm, CNV area of 5.00 mm^2^, and clinical assessment score of 8.4. As for the Fer‐1‐NPs group, the results showed a significant decrease in the CNV length (0.65 mm), CNV area (2.99 mm^2^), and clinical assessment score (6.6) compared to the saline group and the Fer‐1 group.

The gross view of Fer‐1‐NPs‐treated corneas showed a substantial improvement compared to the saline group and the Fer‐1 group, with the background pattern more clearly visible (Figure 4e). Similarly, a notable reduction in infiltrated inflammatory cells and CNV was found in the Fer‐1‐NPs group in H&E‐stained section (Figure 4e). Moreover, Fer‐1‐NPs treatment further reduced corneal edema (central corneal thickness) compared with Fer‐1 treatment (Figure 4f). These findings indicate that liposomes might provide a valid way to deliver Fer‐1 into the alkali‐burned cornea, thus alleviating inflammation and neovascularization more effectively.

Regarding the improved therapeutic effect of Fer‐1‐NPs, when encapsulated in liposomes, the hydrophobic Fer‐1 was suspected to increase solubility and disperse in tears more adequately instead of being cleared quickly. The Dex and Dex‐NPs groups served as positive controls because steroids were well‐established clinically for the treatment of alkali burn. In our results, the Dex group showed a remarkable therapeutic effect on CNV length (0.67 mm) and CNV area (3.02 mm^2^), with a mean clinical assessment score of 7.7, which was not significantly different from that in the Fer‐1‐NPs group. Similar results were found regarding the gross view and H&E‐stained sections. However, unexpectedly, six of 10 mice in the Dex‐NPs group presented corneal perforation on day 14. The mean clinical assessment score of the group was 9.8, indicating that the injuries were at least as severe as those in the saline group. The gross view also showed atrophy and synechia of the cornea (Figure 4e). We speculated that liposomal entrapment enhanced the bioavailability of Dex, thereby mimicking the long‐term use of corticosteroids. Clinically, it could compromise the corneal integrity and increase the risk of corneal melting if the topical administration of corticosteroids lasts for 2 weeks after an alkali burn,^51^ which is consistent with our results. Moreover, the administration of Dex could also have unsatisfactory side effects, including secondary glaucoma, cataracts, and infectious keratitis.^52^ Taken together, Dex could ameliorate alkali burn‐induced corneal injury to an extent equivalent to that of Fer‐1‐NPs, but it also increases the risk of ocular complications. Hence, we excluded Dex and Dex‐NPs therapy in our further experiments.

### 3.5 Possible mechanisms of action of Fer‐1‐NPs on alkali burn

To examine the role that Fer‐1‐NPs play in the alkali burn‐induced ferroptosis, corneal ROS stress production was directly detected by CellROX Green staining according to the previous study.^24^ NADPH oxidase inhibitor DPI was added for comparison. We found that alkali burn treatment significantly increased corneal ROS stress; by contrast, DPI, Fer‐1, and Fer‐1‐NPs all rescued the alkali burn‐induced ROS stress in corneas, while with the best effect in the Fer‐1‐NPs group (Figure S3). These results suggest that Fer‐1‐NPs may function as a more potent radical trap in the treatment of alkali‐burned corneas.

**FIGURE 5:**
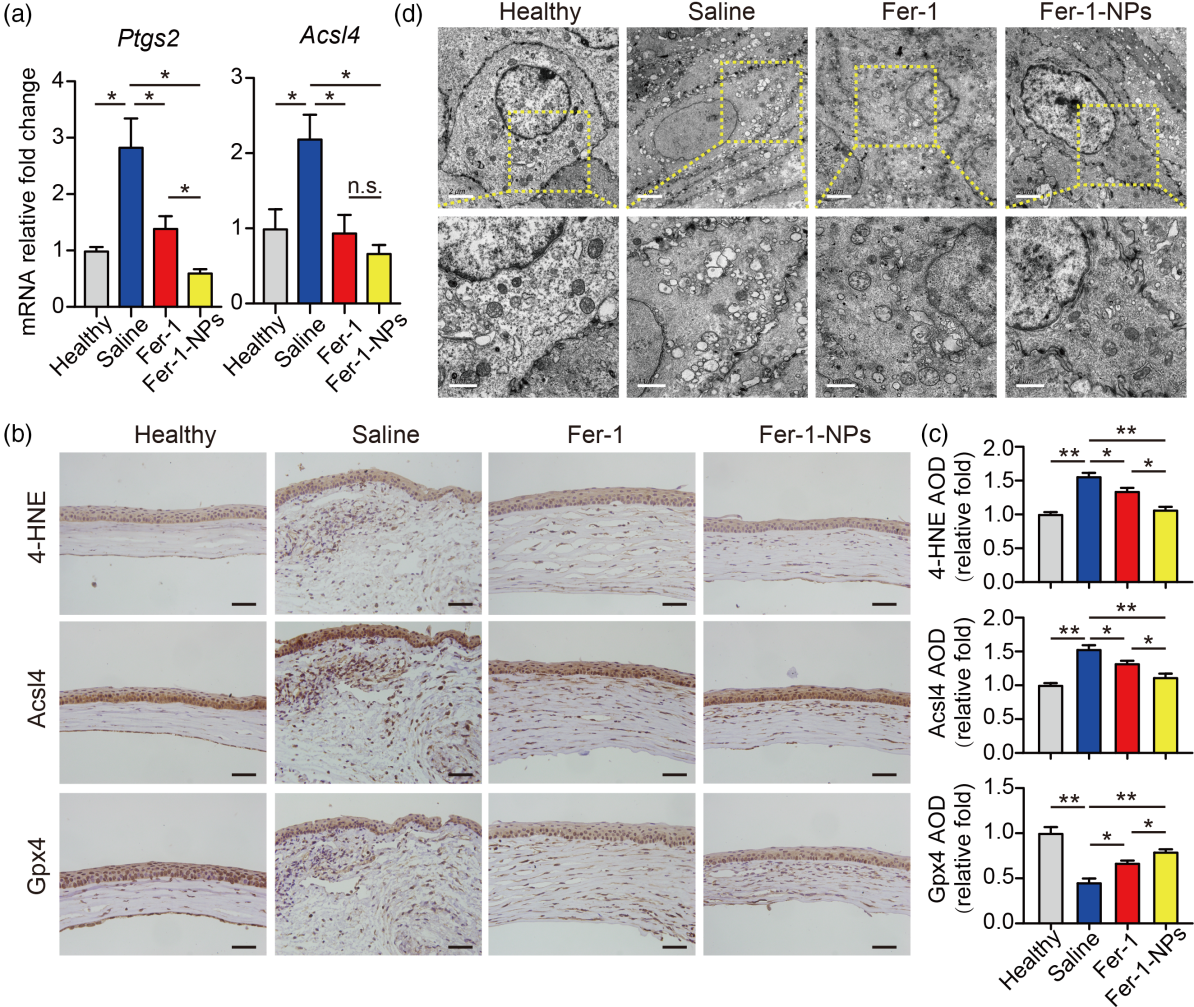
Fer‐1‐NPs exhibited more potent anti‐ferroptosis effects than free Fer‐1. (a) Normalized *Ptgs2* and *Acsl4* mRNA were measured in the indicated groups (*n* = 3). Representative immunohistochemistry images (b) and quantitative summary (c) of corneal sections stained with 4‐HNE (top), Acsl4 (middle), or Gpx4 (bottom); scale bars, 50 μm; *n* = 3. (d) Transmission electron microscope images showing the morphology of mitochondria in corneal tissue obtained from the healthy, saline‐treated, 200 μM Fer‐1‐treated, and 200 μM Fer‐1‐NPs‐treated groups; scale bars represent 2 μm for top images and 1 μm for bottom images. Results were presented as the mean ± *SEM*. *p < 0.05, **p < 0.01, and n.s. stands for not statistically significant. Significance was calculated by one‐way analysis of variance. AOD, average optical density; Fer‐1‐NPs, ferrostatin‐1‐loaded liposomes; 4‐HNE, 4‐hydroxynonenal

To further clarify the mechanisms of Fer‐1‐NPs in the treatment of alkali‐burned corneas via targeting ferroptosis, regulatory genes on the ferroptosis pathway were determined after Fer‐1‐NPs administration. As shown in Figure 5a, compared with Fer‐1, the effect of Fer‐1‐NPs was more potent with respect to rescuing the mRNA expression of *Ptgs2* and *Acsl4*. Consistent with the property of potent radical trap for Fer‐1‐NPs, the lipid peroxide 4‐HNE was most significantly reduced in the Fer‐1‐NPs group (Figure 5b,c). Notably, in addition to the ungoverned protein levels of Gpx4, alkali burn caused a significant accumulation of Acsl4, both of which were almost fully recovered by Fer‐1‐NPs treatment (Figure 5b,c). Acsl4 is a crucial regulatory protein in lipid metabolism. It changes the membrane lipid composition by promoting the oxidation of polyunsaturated fatty acids, thereby sensitizing cells to ferroptosis.^53^ Our results suggest that Acsl4 is involved in the process of corneal injury caused by ferroptosis, and Fer‐1‐NPs may improve corneal injury by inhibiting this pathway. Based on the results above, in alkali burn‐induced corneal injury, the induction pathway of ferroptosis is activated, while the inhibition pathway is attenuated, resulting in ferroptotic cell death in the cornea. Moreover, we found that Fer‐1‐NPs have significantly greater therapeutic effects than free Fer‐1 on restoring the expression of ferroptosis regulators and improving corneal injury caused by ferroptosis. Taken together, these results demonstrate that Fer‐1‐NPs play an important role in the treatment of alkali burn‐induced ferroptosis in corneas by effectively preventing corneal lipid peroxides production.

As for mitochondria morphology, although Fer‐1 treated corneas were somewhat recovered as the shape was more regular and cristae were more abundant than those in the saline group, nevertheless, Fer‐1‐NPs treatment showed a further improvement in revising the mitochondrial damage. These results suggest that Fer‐1‐NPs may play a therapeutic role by remodeling mitochondrial morphology (Figure 5d), indicating a potential effect of Fer‐1‐NPs on maintaining mitochondrial function.

Mitochondrial membrane potential (ΔΨm) is commonly used to detect mitochondrial function, with a loss of ΔΨm indicating mitochondrial depolarization. The presence of an electrochemical potential gradient in functional mitochondria drives accumulation of the JC‐1 fluorescent dye in the mitochondrial matrix, forming red fluorescent (JC‐1 aggregates) in healthy cells; while loss of ΔΨm leading to a fluorescence shift to green one (JC‐1 monomers).^42^ Based on this, we found the red/green fluorescence intensity ratio was decreased obviously in alkali‐burned corneal cells compared with the healthy control (Figure S4), indicating the alkali‐burned injury greatly lowered the ΔΨm of corneal cells. The reduced ΔΨm triggered mitochondria depolarization and dysfunction. Notably, both Fer‐1 and Fer‐1‐NPs rescued this kind of alkali burn‐induced mitochondria depolarization, with a more effective role from Fer‐1‐NPs. This was presumably attributed to the distinguishable effects on maintaining ΔΨm of Fer‐1‐NPs. Thus, our results suggest that Fer‐1‐NPs exert a better therapeutic role by protecting mitochondrial morphology and function.

**FIGURE 6:**
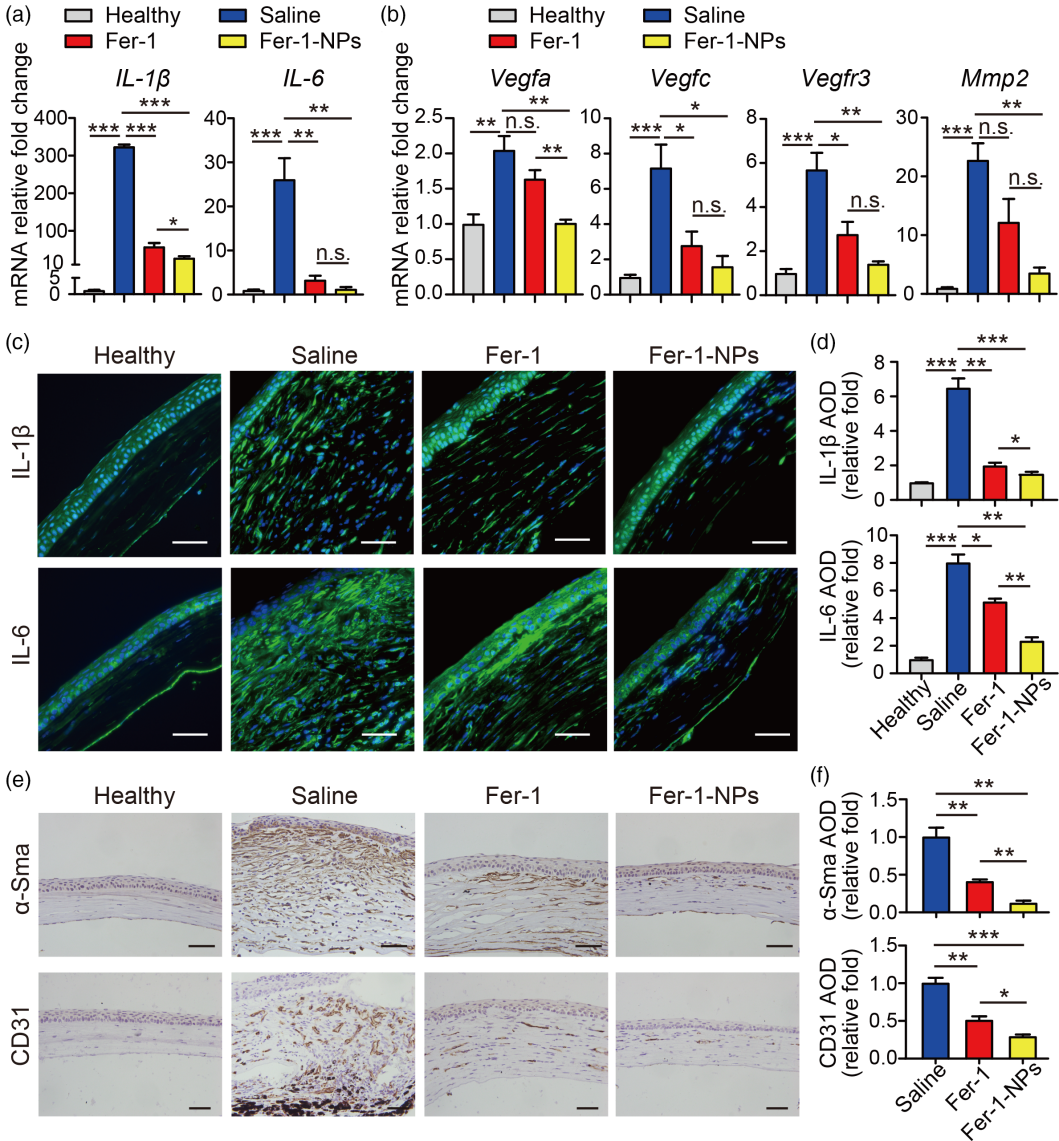
Fer‐1‐NPs exhibited more potent anti‐inflammatory and anti‐angiogenic effects than free Fer‐1. Expression of the indicated inflammatory‐related (a) and angiogenic‐related (b) genes was measured using real‐time polymerase chain reaction in corneal tissue (*n* = 3). Representative immunofluorescence images (c) and quantitative summary (d) of corneal sections stained with IL‐1β (top) or IL‐6 (bottom); scale bars, 50 μm; *n* = 3. Representative immunohistochemistry images (e) and quantitative summary (f) of corneal sections stained with α‐Sma (top) or CD31 (bottom); scale bars, 50 μm; *n* = 3. Results were presented as the mean ± *SEM*. *p < 0.05, **p < 0.01, ***p < 0.001, and n.s. stands for not statistically significant. Significance was calculated by one‐way analysis of variance. AOD, average optical density; Fer‐1‐NPs, ferrostatin‐1‐loaded liposomes

Inflammation is crucial in the pathological progress of corneal alkali burn. Once stimulated by alkali burn, inflammatory cells (e.g., white blood cells) and mesenchymal cells (e.g., macrophages, myofibroblasts) can be activated. In addition, the expression of a large number of cytokines, such as IL‐1β, IL‐6, and IL‐10 in the cornea will be significantly increased, promoting inflammation and the formation of CNV.^54^ Previous studies have suggested that anti‐ROS therapy could effectively alleviate corneal inflammation and inhibit CNV.^24^, ^25^, ^26^, ^27^ To determine whether Fer‐1‐NPs can exert a therapeutic effect by inhibiting inflammation, we tested the mRNA expression levels of IL‐1β and IL‐6. As shown in Figure 6a, corneas treated with Fer‐1‐NPs significantly rescued the high expression of IL‐1β (*p* < 0.001 vs. saline, *p* < 0.05 vs. Fer‐1) and IL‐6 (*p* < 0.01 vs. saline, *p* > 0.05 vs. Fer‐1) in response to alkali‐burned injury. Similar results were found for the IL‐1β and IL‐6 protein levels by immunofluorescence (Figure 6c,d). These results indicate that Fer‐1‐NPs could improve corneal injury by reducing the inflammatory response caused by alkali burn.

Neovascularization is another important pathological feature of corneal alkali burn. As an important factor affecting the visual quality of patients and the prognosis of corneal transplantation, CNV has become a topic of interest in the field of alkali burn. We observed that Fer‐1‐NPs could significantly inhibit the growth of CNV after alkali burn in mice. To explore the anti‐angiogenesis mechanism of Fer‐1‐NPs, we examined the mRNA expression levels of neovascularization‐related genes. As shown in Figure 6b, the Fer‐1‐NPs group had reduced expression of *Vegfa* (*p* < 0.01 vs. saline, *p* < 0.01 vs. Fer‐1) compared to the alkali‐burned groups. A significant decrease in *Vegfc*, *Vegfr3*, and *Mmp2* mRNA levels was also found in corneas treated with Fer‐1‐NPs compared with those in the saline group. VEGFA could facilitate the migration of vascular endothelial cells and increase vascular permeability, which is vital to the growth of pathological neovascularization.^55^, ^56^ Moreover, VEGFC could promote the regeneration of lymphatic vessels by activating VEGFR3.^57^ MMP‐2 is known to provide growth space for CNV by degrading the extracellular matrix.^58^ Meanwhile, pathological neovascularization is also closely related to corneal stroma fibrosis. Therefore, α‐Sma (myofibroblasts marker) and CD31 (vascular endothelial cells marker) are generally considered tissue indicators of CNV.^21^ Our immunohistochemical analyses show that, in the Fer‐1‐NPs group, the expressions of both α‐Sma and CD31 were markedly downregulated compared to the other two groups (Figure 6e,f), suggesting the potent anti‐CNV effect of Fer‐1‐NPs in vivo.

To further demonstrate the anti‐angiogenesis effect of Fer‐1‐NPs, we carried out a VEGF‐induced migration assay and a tube formation assay in vitro. Figures S5 and S6 show that HUVEC‐GFP migration was induced by VEGF, with the maximum healing rate among all treatments. However, a significantly decreased healing rate was induced by Fer‐1 and Fer‐1‐NPs. Remarkably, the healing rate in the cells treated with Fer‐1‐NPs was notably less than that in the cells treated with Fer‐1. Regarding the Matrigel tube formation assay, we observed a similar trend. The tube formation was strikingly decreased when cells were exposed to Fer‐1 or Fer‐1‐NPs, as quantified by the number of junctions. In addition, Fer‐1‐NPs treatment led to a more significant reduction of tube junctions than that of Fer‐1 treatment. The stronger inhibitory effect of Fer‐1‐NPs might be attributed to the enhanced intracellular uptake of liposomes,^59^ which was also demonstrated in our cellular uptake experiment (Figure 3e). Therefore, these results demonstrate that both Fer‐1 and Fer‐1‐NPs could efficiently inhibit VEGF‐induced angiogenesis, offering the important potential for the development of ferroptosis‐targeted anti‐CNV treatment in the clinic.

The anti‐angiogenesis effect by targeting ferroptosis warrants consideration. ROS was reported to act as second messengers in triggering inflammation through the activation of nuclear factor‐κB (NF‐κB), and subsequently stimulate the release of inflammatory cytokines,^60^ the expression of VEGF,^61^ and MMPs,^62^ suggesting that the anti‐angiogenesis effect of Fer‐1 may be due to the reduced inflammation signaling from the relived ROS stress by this potent ferroptosis inhibitor. Moreover, increasing evidence supported that ferroptosis plays an important role as the trigger for initiating inflammation in the injury of liver,^63^ intestine,^64^ brain,^65^ heart,^66^ and renal,^67^ which implicated in several signaling pathways including NF‐κB, TGF‐beta, Nrf2/HO1 and TLR. More pathway analyses are needed in further study to characterize the underlying mechanism of the anti‐angiogenesis effect in alkali‐burned injury by targeting ferroptosis.

### 3.6 Biocompatibility evaluation of Fer‐1‐NPs

**FIGURE 7:**
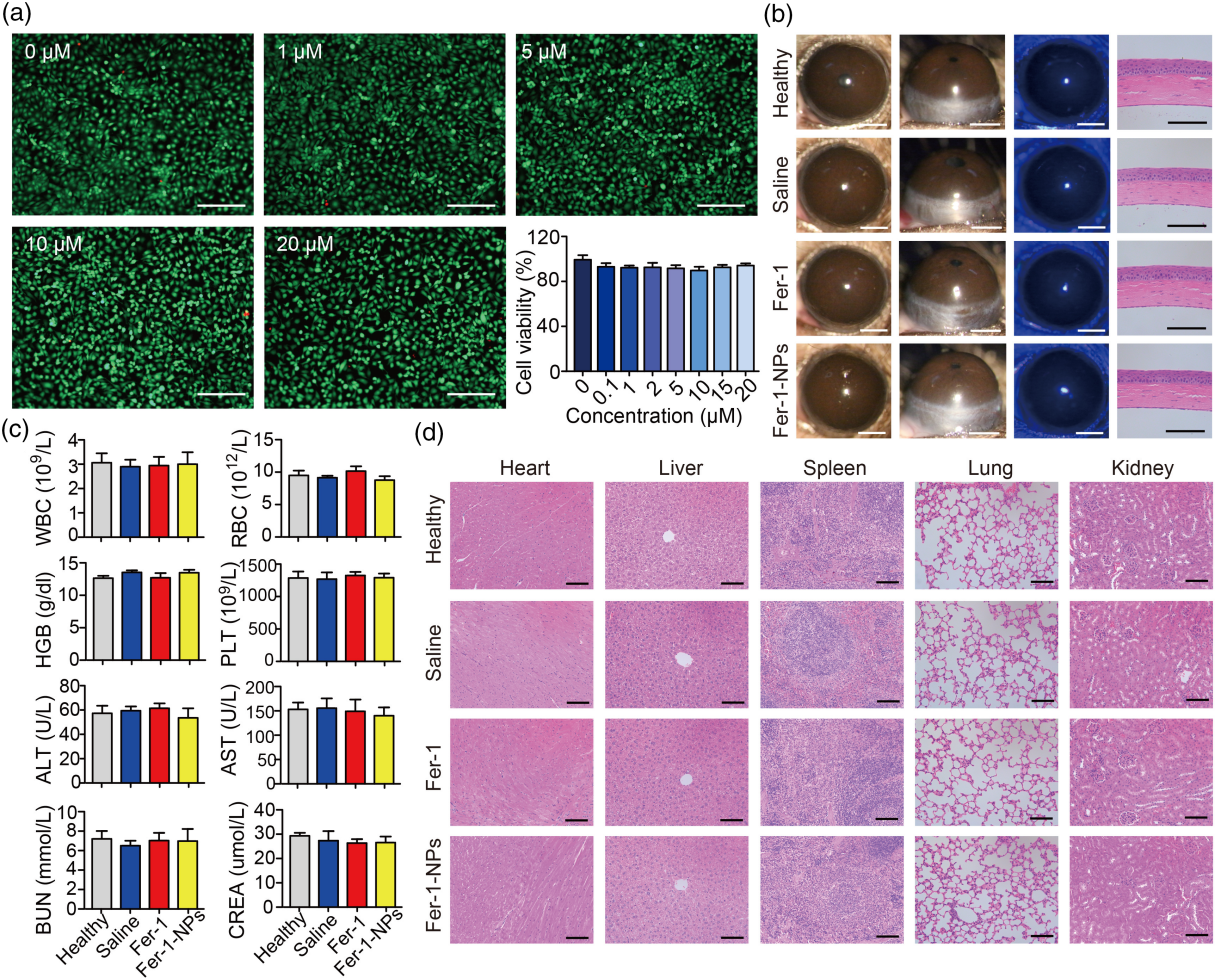
Biocompatibility evaluation of the Fer‐1‐NPs. (a) The live/dead (calcein‐AM/PI) assay and the CCK‐8 assay (*n* = 3) of the human corneal epithelial cells treated with different concentrations of Fer‐1‐NPs were used to measure cell viability in vitro; scale bars, 250 μm. (b) For physiological conditions (without alkali burn), in vivo biocompatibility of no administration (healthy), saline‐treated, 200 μM Fer‐1‐treated, and 200 μM Fer‐1‐NPs‐treated groups were evaluated by slit‐lamp examination, fluorescein sodium staining, and hematoxylin–eosin (H&E) staining on day 14; scale bars represent 1 mm for slit‐lamp images (white) and 100 μm for H&E staining images (black). For pathological conditions (with alkali burn), in vivo biocompatibility of the indicated groups was evaluated by blood routine examination analyses and blood biochemistry (c) and H&E staining of main visceral organs (d); scale bars, 100 μm; *n* = 3. Results were presented as the mean ± *SEM*. ALT, alanine transferase; AST, aspartate transferase; BUN, blood urea nitrogen; CREA, creatinine; Fer‐1‐NPs, ferrostatin‐1‐loaded liposomes; HGB, hemoglobin; PLT, blood platelet; RBC, red blood cells; WBC, white blood cells

The cytotoxicity of the Fer‐1‐NPs was evaluated using calcein‐AM/PI and CCK‐8 assays (Figure 7a). Based on the live/dead cell staining, the majority of HCECs treated with Fer‐1‐NPs displayed green fluorescence. Accordingly, the CCK‐8 assay showed that the cell viability still exceeded 90%, even when the concentration of Fer‐1‐NPs reached 20 μM, which indicated little inhibitory effect on cell proliferation.

The in vivo biocompatibility of Fer‐1‐NPs was evaluated by corneal stimulation assessment in normal mouse eyes (without alkali burn) treated with different formulations, followed by corneal examination using a slit‐lamp microscope (Figure 7b). After administration of Fer‐1‐NPs for 14 consecutive days, there was no evidence of corneal opacity, CNV, inflammation, or hyperemia in the treated corneas. In addition, the integrity of the corneal epithelium was evaluated by fluorescein staining, which revealed no visible green staining, indicating that the corneal epithelium was intact without observable impairment during the Fer‐1‐NPs therapy. Furthermore, H&E staining, which was used to examine corneal anatomical morphology, showed that the corneas in each group maintained a regular appearance, with a tight and orderly arrangement, but without inflammatory cells or CNV.

In addition to physiological conditions, we also investigated the toxicity of Fer‐1‐NPs in pathological conditions (with alkali burn). As shown in Figure 7c, the routine blood parameters as well as liver and renal function were within the normal ranges, without intergroup differences. Moreover, H&E staining of the main visceral organs (heart, liver, spleen, lung, and kidney) showed that the Fer‐1‐NPs treatment did not exhibit noticeable histological changes (Figure 7d). Taken together, no obvious toxic effects and excellent biocompatibility of Fer‐1‐NPs were verified, laying the foundation for clinical application.

## 4 CONCLUSIONS

In this study, Fer‐1‐loaded liposomes, Fer‐1‐NPs were prepared via the film hydration method. The solubility, as well as the precorneal retention time of encapsulated Fer‐1, could be improved, resulting in enhanced bioavailability. An improved therapeutic effect of alkali burn was achieved by Fer‐1‐NPs in the mouse model due to the inhibition of ferroptosis, inflammation, and neovascularization. Moreover, the suppression of angiogenesis by Fer‐1‐NPs was verified by a VEGF‐induced endothelial cell migration assay and a tube formation assay. The Fer‐1‐NPs exhibited no evident cytotoxicity, ocular surface damage, or systemic toxicity. Overall, the Fer‐1‐encapsulated liposomes investigated here could be an efficacious, simple, and safe alternative treatment for corneal alkali burn in the future. Although ferroptosis is a new target with respect to the alkali‐burned injury treatment, combination therapy is worth to be explored. Further studies are necessary to develop co‐encapsulating therapies for alkali‐burned injury treatment via targeting ferroptosis.

## CONFLICT OF INTEREST

The authors have no conflicts of interest to declare.

## AUTHOR CONTRIBUTIONS

**Kai Wang:** Conceptualization (equal); data curation (equal); formal analysis (equal); investigation (equal); methodology (equal); validation (equal); visualization (equal); writing – original draft (equal); writing – review and editing (equal). **Li Jiang:** Conceptualization (equal); data curation (equal); formal analysis (equal); funding acquisition (equal); investigation (equal); methodology (equal); resources (equal); visualization (equal); writing – original draft (equal); writing – review and editing (equal). **Yueyang Zhong:** Data curation (equal); formal analysis (equal); investigation (equal); methodology (equal); validation (equal); visualization (equal); writing – original draft (equal); writing – review and editing (equal). **Yin Zhang:** Data curation (equal); formal analysis (equal); investigation (equal); methodology (equal); validation (equal); writing – original draft (equal); writing – review and editing (equal). **Qichuan Yin:** Formal analysis (equal); investigation (equal); methodology (equal); validation (equal); writing – review and editing (equal). **Su Li:** Formal analysis (equal); investigation (equal); methodology (equal); validation (equal); writing – review and editing (equal). **Xiaobo Zhang:** Formal analysis (equal); investigation (equal); validation (equal); writing – review and editing (equal). **Haijie Han:** Conceptualization (equal); formal analysis (equal); funding acquisition (equal); investigation (equal); project administration (equal); supervision (equal); validation (equal); visualization (equal); writing – original draft (equal); writing – review and editing (equal). **Ke Yao:** Conceptualization (equal); funding acquisition (equal); project administration (equal); resources (equal); supervision (equal); writing – review and editing (equal).

### PEER REVIEW

The peer review history for this article is available at https://publons.com/publon/10.1002/btm2.10276.

## Supporting information


**Data S1.** Supporting information.

## Data Availability

The data that support the findings of this study are available from the corresponding author upon reasonable request.

## References

[1] Sharma N , Kaur M , Agarwal T , Sangwan VS , Vajpayee RB . Treatment of acute ocular chemical burns. Surv Ophthalmol. 2018;63(2):214‐235. 28935121 10.1016/j.survophthal.2017.09.005

[2] Bachmann B , Taylor RS , Cursiefen C . Corneal neovascularization as a risk factor for graft failure and rejection after keratoplasty: an evidence‐based meta‐analysis. Ophthalmology. 2010;117(7):1300‐1305 e1307. 20605214 10.1016/j.ophtha.2010.01.039

[3] Dave RS , Goostrey TC , Ziolkowska M , Czerny‐Holownia S , Hoare T , Sheardown H . Ocular drug delivery to the anterior segment using nanocarriers: a mucoadhesive/mucopenetrative perspective. J Control Release. 2021;336:71‐88. 34119558 10.1016/j.jconrel.2021.06.011

[4] Mandal A , Pal D , Agrahari V , Trinh HM , Joseph M , Mitra AK . Ocular delivery of proteins and peptides: challenges and novel formulation approaches. Adv Drug Deliv Rev. 2018;126:67‐95. 29339145 10.1016/j.addr.2018.01.008 PMC5995646

[5] Singh M , Bharadwaj S , Lee KE , Kang SG . Therapeutic nanoemulsions in ophthalmic drug administration: concept in formulations and characterization techniques for ocular drug delivery. J Control Release. 2020;328:895‐916. 33069743 10.1016/j.jconrel.2020.10.025

[6] Han H , Hou Y , Chen X , et al. Metformin‐induced stromal depletion to enhance the penetration of gemcitabine‐loaded magnetic nanoparticles for pancreatic cancer targeted therapy. J Am Chem Soc. 2020;142(10):4944‐4954. 32069041 10.1021/jacs.0c00650

[7] Han H , Valdeperez D , Jin Q , et al. Dual enzymatic reaction‐assisted gemcitabine delivery systems for programmed pancreatic cancer therapy. ACS Nano. 2017;11(2):1281‐1291. 28071891 10.1021/acsnano.6b05541

[8] Wu D , Zhao Z , Kim J , et al. Gemcitabine and doxorubicin in immunostimulatory monophosphoryl lipid a liposomes for treating breast cancer. Bioeng Transl Med. 2021;6(1):e10188. 33532588 10.1002/btm2.10188 PMC7823124

[9] Zhang J , Li Y , Xiong J , et al. Delivery of pOXR1 through an injectable liposomal nanoparticle enhances spinal cord injury regeneration by alleviating oxidative stress. Bioact Mater. 2021;6(10):3177‐3191. 33778197 10.1016/j.bioactmat.2021.03.001 PMC7970014

[10] Joseph A , Liao R , Zhang M , et al. Nanoparticle‐microglial interaction in the ischemic brain is modulated by injury duration and treatment. Bioeng Transl Med. 2020;5(3):e10175. 33005740 10.1002/btm2.10175 PMC7510458

[11] Han H , Gao Y , Chai M , et al. Biofilm microenvironment activated supramolecular nanoparticles for enhanced photodynamic therapy of bacterial keratitis. J Control Release. 2020;327:676‐687. 32920078 10.1016/j.jconrel.2020.09.014

[12] Janagam DR , Wu L , Lowe TL . Nanoparticles for drug delivery to the anterior segment of the eye. Adv Drug Deliv Rev. 2017;122:31‐64. 28392306 10.1016/j.addr.2017.04.001 PMC6057481

[13] Eriksen AZ , Eliasen R , Oswald J , et al. Multifarious biologic loaded liposomes that stimulate the mammalian target of rapamycin signaling pathway show retina neuroprotection after retina damage. ACS Nano. 2018;12(8):7497‐7508. 30004669 10.1021/acsnano.8b00596 PMC6117751

[14] Filipczak N , Pan J , Yalamarty SSK , Torchilin VP . Recent advancements in liposome technology. Adv Drug Deliv Rev. 2020;156:4‐22. 32593642 10.1016/j.addr.2020.06.022

[15] Fan W , Han H , Chen Y , et al. Antimicrobial nanomedicine for ocular bacterial and fungal infection. Drug Deliv Transl Res. 2021;11(4):1352‐1375. 33840082 10.1007/s13346-021-00966-x

[16] Nishimura T , Hirose S , Sasaki Y , Akiyoshi K . Substrate‐sorting nanoreactors based on permeable peptide polymer vesicles and hybrid liposomes with synthetic macromolecular channels. J Am Chem Soc. 2020;142(1):154‐161. 31766845 10.1021/jacs.9b08598

[17] Lai S , Wei Y , Wu Q , et al. Liposomes for effective drug delivery to the ocular posterior chamber. J Nanobiotechnology. 2019;17(1):64. 31084611 10.1186/s12951-019-0498-7 PMC6515668

[18] Cheng KJ , Hsieh CM , Nepali K , Liou JP . Ocular disease therapeutics: design and delivery of drugs for diseases of the eye. J Med Chem. 2020;63(19):10533‐10593. 32482069 10.1021/acs.jmedchem.9b01033

[19] Chang JH , Gabison EE , Kato T , Azar DT . Corneal neovascularization. Curr Opin Ophthalmol. 2001;12(4):242‐249. 11507336 10.1097/00055735-200108000-00002

[20] Yang J , Luo L , Oh Y , et al. Sunitinib malate‐loaded biodegradable microspheres for the prevention of corneal neovascularization in rats. J Control Release. 2020;327:456‐466. 32822742 10.1016/j.jconrel.2020.08.019 PMC8105765

[21] Han H , Yin Q , Tang X , et al. Development of mucoadhesive cationic polypeptide micelles for sustained cabozantinib release and inhibition of corneal neovascularization. J Mater Chem B. 2020;8(23):5143‐5154. 32420566 10.1039/d0tb00874e

[22] Bakunowicz‐Lazarczyk A , Urban B . Assessment of therapeutic options for reducing alkali burn‐induced corneal neovascularization and inflammation. Adv Med Sci. 2016;61(1):101‐112. 26651127 10.1016/j.advms.2015.10.003

[23] Rama P , Matuska S , Paganoni G , Spinelli A , De Luca M , Pellegrini G . Limbal stem‐cell therapy and long‐term corneal regeneration. N Engl J Med. 2010;363(2):147‐155. 20573916 10.1056/NEJMoa0905955

[24] Gu XJ , Liu X , Chen YY , et al. Involvement of NADPH oxidases in alkali burn‐induced corneal injury. Int J Mol Med. 2016;38(1):75‐82. 27221536 10.3892/ijmm.2016.2594 PMC4899027

[25] Kubota M , Shimmura S , Kubota S , et al. Hydrogen and N‐acetyl‐L‐cysteine rescue oxidative stress‐induced angiogenesis in a mouse corneal alkali‐burn model. Invest Ophthalmol Vis Sci. 2011;52(1):427‐433. 20847117 10.1167/iovs.10-6167

[26] Wan SS , Pan YM , Yang WJ , Rao ZQ , Yang YN . Inhibition of EZH2 alleviates angiogenesis in a model of corneal neovascularization by blocking FoxO3a‐mediated oxidative stress. FASEB J. 2020;34(8):10168‐10181. 32562311 10.1096/fj.201902814RRR

[27] Cejka C , Kossl J , Holan V , Zhang JH , Cejkova J . An Immunohistochemical study of the increase in antioxidant capacity of corneal epithelial cells by molecular hydrogen, leading to the suppression of alkali‐induced oxidative stress. Oxid Med Cell Longev. 2020;2020:7435260. 32655773 10.1155/2020/7435260 PMC7327556

[28] Neitemeier S , Jelinek A , Laino V , et al. BID links ferroptosis to mitochondrial cell death pathways. Redox Biol. 2017;12:558‐570. 28384611 10.1016/j.redox.2017.03.007 PMC5382034

[29] Cejkova J , Trosan P , Cejka C , et al. Suppression of alkali‐induced oxidative injury in the cornea by mesenchymal stem cells growing on nanofiber scaffolds and transferred onto the damaged corneal surface. Exp Eye Res. 2013;116:312‐323. 24145108 10.1016/j.exer.2013.10.002

[30] Dixon SJ , Lemberg KM , Lamprecht MR , et al. Ferroptosis: an iron‐dependent form of nonapoptotic cell death. Cell. 2012;149(5):1060‐1072. 22632970 10.1016/j.cell.2012.03.042 PMC3367386

[31] Stockwell BR , Friedmann Angeli JP , Bayir H , et al. Ferroptosis: a regulated cell death nexus linking metabolism, redox biology, and disease. Cell. 2017;171(2):273‐285. 28985560 10.1016/j.cell.2017.09.021 PMC5685180

[32] Linkermann A , Skouta R , Himmerkus N , et al. Synchronized renal tubular cell death involves ferroptosis. Proc Natl Acad Sci U S A. 2014;111(47):16836‐16841. 25385600 10.1073/pnas.1415518111 PMC4250130

[33] Mao L , Zhao T , Song Y , et al. The emerging role of ferroptosis in non‐cancer liver diseases: hype or increasing hope? Cell Death Dis. 2020;11(7):518. 32647111 10.1038/s41419-020-2732-5 PMC7347946

[34] Friedmann Angeli JP , Krysko DV , Conrad M . Ferroptosis at the crossroads of cancer‐acquired drug resistance and immune evasion. Nat Rev Cancer. 2019;19(7):405‐414. 31101865 10.1038/s41568-019-0149-1

[35] Sun Y , Zheng Y , Wang C , Liu Y . Glutathione depletion induces ferroptosis, autophagy, and premature cell senescence in retinal pigment epithelial cells. Cell Death Dis. 2018;9(7):753. 29988039 10.1038/s41419-018-0794-4 PMC6037763

[36] Chen C , Chen J , Wang Y , Liu Z , Wu Y . Ferroptosis drives photoreceptor degeneration in mice with defects in all‐trans‐retinal clearance. J Biol Chem. 2021;296:100187. 33334878 10.1074/jbc.RA120.015779 PMC7948481

[37] Shu W , Baumann BH , Song Y , Liu Y , Wu X , Dunaief JL . Ferrous but not ferric iron sulfate kills photoreceptors and induces photoreceptor‐dependent RPE autofluorescence. Redox Biol. 2020;34:101469. 32362442 10.1016/j.redox.2020.101469 PMC7327978

[38] Lovatt M , Adnan K , Kocaba V , Dirisamer M , Peh GSL , Mehta JS . Peroxiredoxin‐1 regulates lipid peroxidation in corneal endothelial cells. Redox Biol. 2020;30:101417. 31901729 10.1016/j.redox.2019.101417 PMC6948265

[39] Wiraja C , Mathiyazhakan M , Movahedi F , et al. Near‐infrared light‐sensitive liposomes for enhanced plasmid DNA transfection. Bioeng Transl Med. 2016;1(3):357‐364. 29313020 10.1002/btm2.10020 PMC5689532

[40] Chen X , Jia F , Li Y , et al. Nitric oxide‐induced stromal depletion for improved nanoparticle penetration in pancreatic cancer treatment. Biomaterials. 2020;246:119999. 32247201 10.1016/j.biomaterials.2020.119999

[41] Alfaifi AA , Heyder RS , Bielski ER , et al. Megalin‐targeting liposomes for placental drug delivery. J Control Release. 2020;324:366‐378. 32461116 10.1016/j.jconrel.2020.05.033 PMC8247794

[42] Yao X , Ma Y , Zhou W , et al. In‐cytoplasm mitochondrial transplantation for mesenchymal stem cells engineering and tissue regeneration. Bioeng Transl Med. 2021;e10250. Epub ahead of print. 10.1002/btm2.10250 PMC8780934 35111950

[43] Guell JL , El Husseiny MA , Manero F , Gris O , Elies D . Historical review and update of surgical treatment for corneal endothelial diseases. Ophthalmol Ther. 2014;3(1–2):1‐15. 25134494 10.1007/s40123-014-0022-y PMC4254859

[44] Yang WS , SriRamaratnam R , Welsch ME , et al. Regulation of ferroptotic cancer cell death by GPX4. Cell. 2014;156(1–2):317‐331. 24439385 10.1016/j.cell.2013.12.010 PMC4076414

[45] Yamada N , Karasawa T , Kimura H , et al. Ferroptosis driven by radical oxidation of n‐6 polyunsaturated fatty acids mediates acetaminophen‐induced acute liver failure. Cell Death Dis. 2020;11(2):144. 32094346 10.1038/s41419-020-2334-2 PMC7039960

[46] Ingold I , Berndt C , Schmitt S , et al. Selenium utilization by GPX4 is required to prevent hydroperoxide‐induced ferroptosis. Cell. 2018;172(3):409‐422 e421. 29290465 10.1016/j.cell.2017.11.048

[47] Bock FJ , Tait SWG . Mitochondria as multifaceted regulators of cell death. Nat Rev Mol Cell Biol. 2020;21(2):85‐100. 31636403 10.1038/s41580-019-0173-8

[48] Stockwell BR , Jiang X , Gu W . Emerging mechanisms and disease relevance of ferroptosis. Trends Cell Biol. 2020;30(6):478‐490. 32413317 10.1016/j.tcb.2020.02.009 PMC7230071

[49] Cheng R , Li C , Li C , et al. The artemisinin derivative artesunate inhibits corneal neovascularization by inducing ROS‐dependent apoptosis in vascular endothelial cells. Invest Ophthalmol Vis Sci. 2013;54(5):3400‐3409. 23611999 10.1167/iovs.12-11068 PMC5963000

[50] Bhattacharjee S . DLS and zeta potential ‐ what they are and what they are not? J Control Release. 2016;235:337‐351. 27297779 10.1016/j.jconrel.2016.06.017

[51] Donshik PC , Berman MB , Dohlman CH , Gage J , Rose J . Effect of topical corticosteroids on ulceration in alkali‐burned corneas. Arch Ophthalmol. 1978;96(11):2117‐2120. 214063 10.1001/archopht.1978.03910060497024

[52] Tallab RT , Stone DU . Corticosteroids as a therapy for bacterial keratitis: an evidence‐based review of ‘who, when and why’. Br J Ophthalmol. 2016;100(6):731‐735. 26743622 10.1136/bjophthalmol-2015-307955

[53] Doll S , Proneth B , Tyurina YY , et al. ACSL4 dictates ferroptosis sensitivity by shaping cellular lipid composition. Nat Chem Biol. 2017;13(1):91‐98. 27842070 10.1038/nchembio.2239 PMC5610546

[54] Ibrahim Al‐Mashahedah AM , Kanwar RK , Kanwar JR . Utility of nanomedicine targeting scar‐forming myofibroblasts to attenuate corneal scarring and haze. Nanomedicine (Lond). 2019;14(8):1049‐1072. 30901304 10.2217/nnm-2017-0305

[55] Stevenson W , Cheng SF , Dastjerdi MH , Ferrari G , Dana R . Corneal neovascularization and the utility of topical VEGF inhibition: ranibizumab (Lucentis) vs bevacizumab (Avastin). Ocul Surf. 2012;10(2):67‐83. 22482468 10.1016/j.jtos.2012.01.005 PMC3471139

[56] Notara M , Lentzsch A , Coroneo M , Cursiefen C . The role of limbal epithelial stem cells in regulating corneal (lymph)angiogenic privilege and the micromilieu of the limbal niche following UV exposure. Stem Cells Int. 2018;2018:8620172. 29853920 10.1155/2018/8620172 PMC5964490

[57] Martinez‐Corral I , Zhang Y , Petkova M , et al. Blockade of VEGF‐C signaling inhibits lymphatic malformations driven by oncogenic PIK3CA mutation. Nat Commun. 2020;11(1):2869. 32513927 10.1038/s41467-020-16496-y PMC7280302

[58] Wells JM , Gaggar A , Blalock JE . MMP generated matrikines. Matrix Biol. 2015;44–46:122‐129. 10.1016/j.matbio.2015.01.016 PMC4838901 25636538

[59] Un K , Sakai‐Kato K , Oshima Y , Kawanishi T , Okuda H . Intracellular trafficking mechanism, from intracellular uptake to extracellular efflux, for phospholipid/cholesterol liposomes. Biomaterials. 2012;33(32):8131‐8141. 22858002 10.1016/j.biomaterials.2012.07.030

[60] Saika S , Miyamoto T , Yamanaka O , et al. Therapeutic effect of topical administration of SN50, an inhibitor of nuclear factor‐kappaB, in treatment of corneal alkali burns in mice. Am J Pathol. 2005;166(5):1393‐1403. 15855640 10.1016/s0002-9440(10)62357-7 PMC1606394

[61] Zhou AY , Bai YJ , Zhao M , Yu WZ , Li XX . KH902, a recombinant human VEGF receptor fusion protein, reduced the level of placental growth factor in alkali burn induced‐corneal neovascularization. Ophthalmic Res. 2013;50(3):180‐186. 24008241 10.1159/000353437

[62] Carter RT , Kambampati R , Murphy CJ , Bentley E . Expression of matrix metalloproteinase 2 and 9 in experimentally wounded canine corneas and spontaneous chronic corneal epithelial defects. Cornea. 2007;26(10):1213‐1219. 18043179 10.1097/ICO.0b013e31814b8a28

[63] Tsurusaki S , Tsuchiya Y , Koumura T , et al. Hepatic ferroptosis plays an important role as the trigger for initiating inflammation in nonalcoholic steatohepatitis. Cell Death Dis. 2019;10(6):449. 31209199 10.1038/s41419-019-1678-y PMC6579767

[64] Mayr L , Grabherr F , Schwarzler J , et al. Dietary lipids fuel GPX4‐restricted enteritis resembling Crohn's disease. Nat Commun. 2020;11(1):1775. 32286299 10.1038/s41467-020-15646-6 PMC7156516

[65] Ates G , Goldberg J , Currais A , Maher P . CMS121, a fatty acid synthase inhibitor, protects against excess lipid peroxidation and inflammation and alleviates cognitive loss in a transgenic mouse model of Alzheimer's disease. Redox Biol. 2020;36:101648. 32863221 10.1016/j.redox.2020.101648 PMC7394765

[66] Li W , Feng G , Gauthier JM , et al. Ferroptotic cell death and TLR4/Trif signaling initiate neutrophil recruitment after heart transplantation. J Clin Invest. 2019;129(6):2293‐2304. 30830879 10.1172/JCI126428 PMC6546457

[67] Zhao Z , Wu J , Xu H , et al. XJB‐5‐131 inhibited ferroptosis in tubular epithelial cells after ischemia‐reperfusion injury. Cell Death Dis. 2020;11(8):629. 32796819 10.1038/s41419-020-02871-6 PMC7429848

