# Supplementary material for: Ferrostatin‐1‐loaded liposome for treatment of corneal alkali burn via targeting ferroptosis
Source: Bioeng Transl Med. 2021 Dec 8;7(2):e10276. doi: 10.1002/btm2.10276 (PMC9115688; doi:10.1002/btm2.10276)
Supplement: Supplementary file 1 — Data S1. Supporting information. [file BTM2-7-e10276-s001.docx]

**Supporting Information**

**Ferrostatin-1-loaded Liposome for Treatment of Corneal Alkali Burn *via* Targeting Ferroptosis**

Kai Wang ^1,2#^, Li Jiang ^3#^, Yueyang Zhong ^1,2#^, Yin Zhang ^1,2^, Qichuan Yin ^1,2^, Su Li ^1,2^, Xiaobo Zhang ^1,2^, Haijie Han ^1,2*^, Ke Yao ^1,2*^

^1^ Eye Center, the Second Affiliated Hospital, School of Medicine, Zhejiang University, Hangzhou 310009, People’s Republic of China.

^2^ Zhejiang Provincial Key Lab of Ophthalmology, the Second Affiliated Hospital, School of Medicine, Zhejiang University, Hangzhou 310009, People’s Republic of China.

^3^ Department of Biochemistry and Molecular Biology, School of Basic Medical Sciences, Hangzhou Normal University, Hangzhou 311121, People’s Republic of China.

^*^ Correspondence: Ke Yao (E-mail: xlren@zju.edu.cn);

Co-corresponding author: Haijie Han (E-mail: hanhj90@zju.edu.cn).

^#^ These authors contributed equally to this work.

Table of Contents

**Figure S1.** HPLC standard curve for Fer-1 (A) and Dex (B).

Figure S2. The DLS-determined hydrodynamic diameter of the Dex-NPs. Results were presented as the mean ± SD (n = 3).

**Figure S3.** Representative immunofluorescence images (A) and quantitative summary (B) of corneal flat-mount stained with CellROX Green reagent. Green indicates the fluorescence signal of ROS, and blue indicates DAPI; scale bars, 50 µm. Results were presented as the mean ± SEM (n = 3). * *P* < 0.05, ** *P* < 0.01, and *** P < 0.001. Significance was calculated by one-way ANOVA.

**Figure S4.** Representative immunofluorescence images (A) and quantitative summary (B) of corneal sections stained with JC-1. Red indicates the JC-1 aggregates, green indicates the JC-1 monomers, and blue indicates DAPI; scale bars, 50 µm. Results were presented as the mean ± SEM (n = 3). * *P* < 0.05, ** *P* < 0.01, and *** P < 0.001. Significance was calculated by one-way ANOVA.

**Figure S5.** Representative images (A) and quantitative summary (B) of the healing rate of scratch wound migration assay. The HUVECs-GFP were exposed to medium (control) and VEGF (at a final concentration of 200 ng/mL) with or without the treatment of Fer-1 (at a final concentration of 5 µM) or Fer-1-NPs (at a final Fer-1 concentration of 5 µM); scale bars, 100 µm. Results were presented as the mean ± SEM (n = 3). *** P < 0.001. Significance was calculated by one-way ANOVA.

**Figure S6.** Representative images (A) and quantitative summary (B) of the number of junctions of Matrigel tube formation assay. The HUVECs-GFP were exposed to medium (control) and VEGF (at a final concentration of 200 ng/mL) with or without the treatment of Fer-1 (at a final concentration of 5 µM) or Fer-1-NPs (at a final Fer-1 concentration of 5 µM); scale bars, 100 µm. Results were presented as the mean ± SEM (n = 3). * *P* < 0.05 and *** P < 0.001. Significance was calculated by one-way ANOVA.


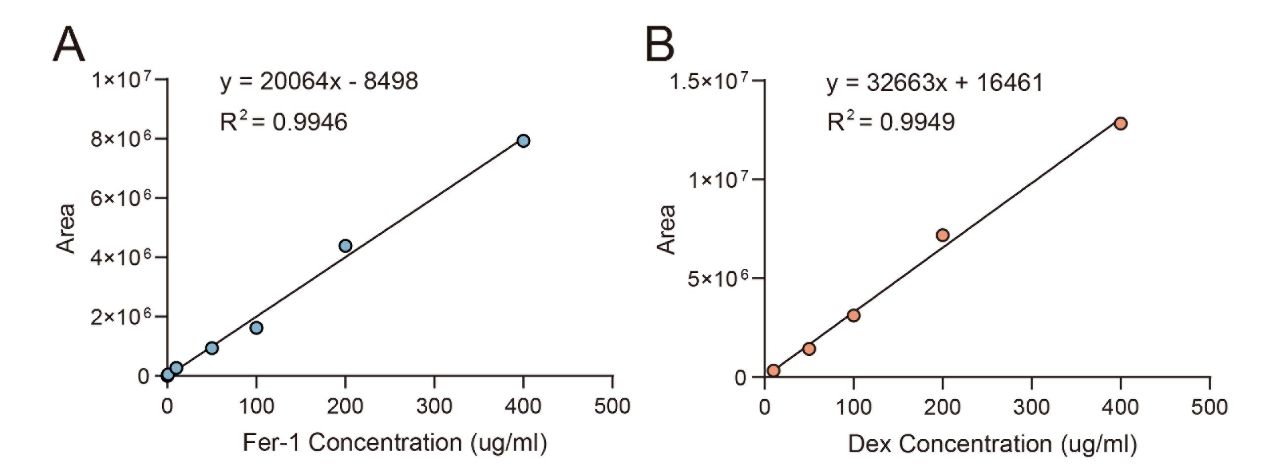


**Figure S1.** HPLC standard curve for Fer-1 (A) and Dex (B).





Figure S2. The DLS-determined hydrodynamic diameter of the Dex-NPs. Results were presented as the mean ± SD (n = 3).





**Figure S3.** Representative immunofluorescence images (A) and quantitative summary (B) of corneal flat-mount stained with CellROX Green reagent. Green indicates the fluorescence signal of ROS, and blue indicates DAPI; scale bars, 50 µm. Results were presented as the mean ± SEM (n = 3). * *P* < 0.05, ** *P* < 0.01, and *** P < 0.001. Significance was calculated by one-way ANOVA.





**Figure S4.** Representative immunofluorescence images (A) and quantitative summary (B) of corneal sections stained with JC-1. Red indicates the JC-1 aggregates, green indicates the JC-1 monomers, and blue indicates DAPI; scale bars, 50 µm. Results were presented as the mean ± SEM (n = 3). * *P* < 0.05, ** *P* < 0.01, and *** P < 0.001. Significance was calculated by one-way ANOVA.





**Figure S5.** Representative images (A) and quantitative summary (B) of the healing rate of scratch wound migration assay. The HUVECs-GFP were exposed to medium (control) and VEGF (at a final concentration of 200 ng/mL) with or without the treatment of Fer-1 (at a final concentration of 5 µM) or Fer-1-NPs (at a final Fer-1 concentration of 5 µM); scale bars, 100 µm. Results were presented as the mean ± SEM (n = 3). *** P < 0.001. Significance was calculated by one-way ANOVA.





**Figure S6.** Representative images (A) and quantitative summary (B) of the number of junctions of Matrigel tube formation assay. The HUVECs-GFP were exposed to medium (control) and VEGF (at a final concentration of 200 ng/mL) with or without the treatment of Fer-1 (at a final concentration of 5 µM) or Fer-1-NPs (at a final Fer-1 concentration of 5 µM); scale bars, 100 µm. Results were presented as the mean ± SEM (n = 3). * *P* < 0.05 and *** P < 0.001. Significance was calculated by one-way ANOVA.
